# Organelle Visualization With Multicolored Fluorescent Markers in Bamboo

**DOI:** 10.3389/fpls.2021.658836

**Published:** 2021-04-15

**Authors:** Mengdi Zhang, Shuai Hu, Fang Yi, Yanli Gao, Dongmei Zhu, Yizhu Wang, Yi Cai, Dan Hou, Xinchun Lin, Jinbo Shen

**Affiliations:** ^1^State Key Laboratory of Subtropical Silviculture, Zhejiang A&F University, Hangzhou, China; ^2^College of Life Science, Sichuan Agricultural University, Ya’an, China

**Keywords:** protoplast transient expression, organelle markers, membrane compartments, subcellular localization, protein–protein interaction, live imaging

## Abstract

Bamboo is an important model plant to study the molecular mechanisms of rapid shoot growth and flowering once in a lifetime. However, bamboo research about protein functional characterization is largely lagged behind, mainly due to the lack of gene transformation platforms. In this study, a protoplast transient gene expression system in moso bamboo has been first established. Using this reliable and efficient system, we have generated a set of multicolored fluorescent markers based on the targeting sequences from endogenous proteins, which have been validated by their comparative localization with *Arabidopsis* organelle markers, in a combination with pharmaceutical treatments. Moreover, we further demonstrated the power of this multicolor marker set for rapid, combinatorial analysis of the subcellular localization of uncharacterized proteins, which may play potential functions in moso bamboo flowering and fast growth of shoots. Finally, this protoplast transient gene expression system has been elucidated for functional analysis in protein–protein interaction by fluorescence resonance energy transfer (FRET) and co-immunoprecipitation analysis. Taken together, in combination with the set of moso bamboo organelle markers, the protoplast transient gene expression system could be used for subcellular localization and functional study of unknown proteins in bamboo and will definitely promote rapid progress in diverse areas of research in bamboo plants.

## Introduction

Bamboo is one of the most crucial non-timber forest plants that can grow in tropical, subtropical, and temperate regions in the world (Tanaka et al., 2003; Peng et al., 2013). Bamboo is symbolized as high yield and fast growth with a long vegetative lifetime, and the other uniqueness of this plant is that it flowers once in a lifetime (Wu et al., 2015). Recently, great research progresses have been achieved on the moso bamboo, *Phyllostachys edulis* (Carrière) J. Houzeau (synonym *P*. *heterocycla* Carrière), in the molecular mechanism of bamboo fast growth and flowering at the genomic or transcriptomic level. For example, the high-quality draft genome sequence and the transcriptomic sequencing data of moso bamboo have been published recently (Peng et al., 2013), and the complete sequence map of its chloroplast genome has also been reported (Zhang Y. J. et al., 2011; Peng et al., 2013). In addition, depending on the integrating high-throughput DNA and RNA sequencing data of moso bamboo, the online analytical platforms with comprehensively functional genome annotation and gene coexpression network have been successfully built (Zhao et al., 2014; Ma et al., 2018), which is now feasible for us to predict the relationships between gene expression and growth traits of moso bamboo. However, very few studies have been explored for protein subcellular localization and functional analysis in bamboo, mainly due to the lack of gene transformation platforms and reliable organelle markers. In this context, the transient protein expression system is a complement for the stable transformation method and should be a useful tool in investigating protein localization and molecular functional studies at the cellular or subcellular level.

The transient gene expression system, either in plant protoplasts or specific tissues, is a powerful tool in cell biology researches in plant systems. Taking the advantage of its convenience, rapidity, and flexibility, the transient gene expression in protoplasts has been the most efficient and widely used method in model plants for various functional studies including subcellular localization (Miao and Jiang, 2007; Yoo et al., 2007; Shen et al., 2014b; Falter et al., 2019), protein–protein interaction (Yang et al., 2014; Hu et al., 2020a; Xiao et al., 2020), investigation of promoters and regulatory elements that are involved in transcription and translation (Jacobsen and Beach, 1985), and high-throughput examination of cell signaling transduction pathways in response to phytohormones, environmental cues, and pathogen-derived elicitors (Assmann et al., 1985; Sheen, 2001; Li et al., 2013; Fraiture et al., 2014; Lin et al., 2014). Due to the versatility and convenience to detect physiological and biochemical responses, protoplast-based transformation systems have also been recently established and applied to non-model plants whose transgenic platforms are not yet available or for which regeneration of transgenic plants is difficult, such as wheat (Hensel et al., 2011), maize (*Zea mays*) (Sheen, 2001), carrot (*Daucus carota*) (Rasmussen and Rasmussen, 1993), perennial ryegrass (*Lolium perenne*) (Yu et al., 2017), pineapple (*Ananas comosus*) (Priyadarshani et al., 2018), and soybean (*Glycine max*) (Xiong et al., 2019). Previously, protoplasts from suspension cells and leaves in several bamboo species have been successfully isolated (Huang et al., 1989; Hisamoto and Kobayashi, 2010; Yeh et al., 2011). Most recently, protoplasts from two bamboo species (*Bambusa oldhamii* and *Dendrocalamus latiflorus*) have been used to evaluate CRISPR-/Cas9-based gene editing reagents (Lin et al., 2018; Ye et al., 2020). Unfortunately, the two studies archived low gene transformation efficiency at around 50%, which have not met the minimum threshold to generate reliable and repeatable data for successful molecular researches (Yoo et al., 2007). Besides, the detailed procedures of protoplast transient expression and its broad usage for protein localization and functional studies have not been rigorously tested.

Understanding the subcellular localization of proteins is quite essential in studying the biological function of enzymes and regulatory proteins within the cells (Millar et al., 2009; Hu et al., 2020b; Wang et al., 2020). Recently, a number of new approaches in studying the subcellular localization of plant proteins have been established, including organelle fractionation, immunofluorescence, immunoelectron microscopy, and expression of fluorescence protein fusion (Tanz et al., 2013; Zhu et al., 2020). Compared with organelle fractionation and immunolocalization, fusion of a fluorescent protein (FP), such as GFP and its derivates, can be used to detect dual or multilocalization and trace dynamics of the proteins of interest in living cells (Geldner et al., 2009; Tanz et al., 2013; Wu et al., 2016; Dangol et al., 2017). Nowadays, the fusion chimeric genes can be incorporated into plant cells via transient expression techniques, which thus has opened up the possibility of directly studying protein subcellular localizations, protein transport dynamics, and protein interactions conveniently (Pendin et al., 2017). Over the past decade, a set of organelle markers containing multicolored fluorescent tags have been developed in *Arabidopsis* and rice and used for co-localization studies with transient expression method (Nelson et al., 2007). Up to now, the subcellular localization of bamboo proteins has been mostly studied in heterologous plants such as *Arabidopsis* and rice leaf protoplasts (Ge et al., 2017; Zhang et al., 2018), onion epidermal cells (Xiao et al., 2018), or tobacco leaves (Liu et al., 2018). However, ectopic expression of proteins in different plant species may result in mis-localization because of the non-conservation of signal sequences or posttranscriptional modifications in the heterologous expression system (Marion et al., 2008). Therefore, a set of organelle markers of endogenous proteins is needed in bamboo, which is compatible with the transient expression system and will provide a versatile system to precisely detect the subcellular localization of bamboo proteins and analyze the dynamics of proteins or organelles upon different physiological or environmental stress conditions.

In this study, we developed a new method for the efficient isolation and gene transformation of protoplasts derived from moso bamboo green seedlings. Next, we identified and expressed multiple fluorescent protein-fused endogenous proteins as organelle markers in moso bamboo protoplasts, and further validated the capability of this multicolored marker set for rapid, combinatorial analysis of plant cell membrane compartments in live-imaging confocal microscopy. Moreover, to test the reliability of the organelle markers in the transient expression system, we characterized the subcellular localizations of several uncharacterized proteins, which may play potential functions in moso bamboo flowering and shoot fast growth, and the results confirmed that this method is reliable and powerful in determining the subcellular of a functional protein. Finally, we demonstrated the power of this transient expression system for protein–protein interaction and protein transport dynamics of moso bamboo in endosomal trafficking.

## Materials and Methods

### Plasmid Construction

The protein sequences of *Arabidopsis* organelle markers were obtained from The Arabidopsis Information Resource^1^. The protein sequences were then used as queries to Blast search against whole protein dataset of moso bamboo in the Bamboo Genome Database^2^. The protein with the highest value was identified as the closest homolog, and the full-length cDNAs encoding the corresponding genes were queried for further cloning. Signal peptide was predicted by SIGNALP v5.0^3^. The transmembrane domain was predicted by TMHMM v2.0^4^.

For the constructs used for transient expression in this study, the cDNAs encoding the corresponding genes or sequences were amplified by PCR and inserted into the premade pBI221 transient expression cassette containing enhanced green fluorescent protein (GFP), monomeric red fluorescent protein (mRFP), or cyan fluorescent protein (CFP)/cerulean fragments under the 35S promoter by restriction digestion. The full-length open reading frames (ORFs) or the compartment-specific targeting signals from moso bamboo genes were generated by PCR, using oligonucleotide primers as given in Supplementary Table 3. All constructs were confirmed by restriction mapping and DNA sequencing.

### Plant Growth Conditions

Moso bamboo seeds were collected from the plants of a single individual growing in Guangxi Province in southern China. Seeds were surface sterilized using 75% (v/v) ethanol for 5 min, and the germinated seedlings were grown hydroponically in a growth chamber with the following environmental settings: 14-h light at ∼500 μmol photons m^–2^ s^–1^ and 10-h dark at 25°C, 60% humidity. Seedlings were grown for 5 weeks for protoplast isolation. For protoplast isolation from micropropagated plantlet, moso bamboo seedlings were cultured in full-strength MS medium. Cultures were maintained under a light intensity of 3,000 lx and a day/night cycle of 8/16 h at 25 ± 2°C in a controlled environment. Micropropagated seedlings were grown for 5–7 weeks for protoplast isolation.

### Protoplast Isolation

Briefly, different tissues including the root, stem, leaf sheath, or leaf blade of seedlings were harvested and rinsed with distilled water to remove any adhered compost. The plant tissue was cut into ∼1-mm slices using a sharp razor blade on filter paper and then transferred quickly and gently submerged into one of the enzyme cocktails described in Supplementary Table 1 for vacuum infiltrate for 30 min in the dark using a desiccator. The samples were then incubated for 4 h in the dark with gentle shaking (40–50 rpm) at 25°C to allow digestion of cell wall materials. After incubation, the plant tissue in digestion solution was shaken gently for 20 s to release the protoplasts followed by filtration through a 50-μm cell strainer to collect the protoplast suspension. The retained tissue was subsequently rinsed with W5 solution (154 mM NaCl, 125 mM CaC1_2_, 5 mM KC1, 2 mM MES, and pH 5.7) with shaking to release the remaining protoplasts followed by filtration. Then, the protoplast suspensions were centrifuged at 150 × *g* for 3 min to collect the protoplasts and then gently washed twice with W5 solution. The pellet was finally resuspended in MMg solution (4 mM MES, pH 5.7, 0.4 M mannitol, and 15 mM MgCl_2_) for quality check and quantified by microscopy using a hemocytometer. The protoplast yield was defined as the total number of protoplasts per fresh leaf mass. The viability of protoplasts was determined by counting the number of protoplasts stained with fluorescein diacetate (FDA, Sigma) under a fluorescence microscope and is expressed as the percentage of fluorescing protoplasts to the total number of protoplasts.

### Protoplast Transformation

The protoplasts were adjusted to a concentration of 5 × 10^5^ protoplasts ml^–1^, and from this, 100 μl of protoplasts was mixed with 10 μl of plasmids (with different concentrations) and 110 μl of polyethylene glycol (PEG) solution (0.2 M mannitol, 0.1 M CaCl_2_, and different concentrations of PEG 4000) in a 2-ml Eppendorf tube. To optimize the transfection duration, the transformation mixture was incubated for 15 min in darkness at 25°C. The transformation process was terminated by the addition of 440 μl of W5 solution followed by gentle mixing. The supernatant was carefully removed after centrifugation at 150 × *g* for 5 min. The protoplast pellet was then re-suspended in 1 ml of W5 solution and finally transferred to multiwell plates. Transformed protoplasts were incubated for 8–12 h at 25°C without shaking. After incubation, protoplasts were pelleted by centrifugation at 150 × *g* for 2 min to remove the supernatant, and protoplasts were re-suspended with the remaining solution. Protoplasts were observed and imaged under a Zeiss Axio Imager.A2 fluorescence microscope. Transfection efficiency was calculated as the percentage of fluorescing protoplasts to total protoplasts.

### Transient Expression in *Arabidopsis* Mesophyll Protoplasts

The transient gene expression system using *Arabidopsis* mesophyll protoplasts was performed as described before (Yoo et al., 2007). The protoplasts were incubated for 8–12 h prior to confocal imaging analysis.

### Confocal Laser Scanning Microscopy and FRET Analysis

Confocal fluorescence imaging was performed 8–12 h after transformation using Leica TCS SP8 or Zeiss LSM 880 Confocal Laser Scanning Microscope as previously described (Shen et al., 2018). Briefly, confocal fluorescence images were acquired using the sequential acquisition mode with a ×63 water lens. A sequential acquisition was applied when observing these fluorescent proteins. Images were processed using the Adobe Photoshop software^5^. For each experiment or construct, more than 30 individual cells were observed for confocal imaging that represented >75% of the samples showing similar expression levels and patterns.

The FRET acceptor bleaching analysis was basically conducted on Leica SP8 confocal system according to the manufacturer’s instruction. Cerulean/CFP donor fluorescence was imaged before and after bleaching a region of interest of EYFP to <10% of its initial intensity. FRET efficiency was calculated as Ef = 100 × (Ipost − Ipre)/Ipost, where Ipre and Ipost stand for the cerulean intensities before and after acceptor bleaching, respectively.

### Co-immunoprecipitation and Immunoblot Analysis

To perform co-immunoprecipitation experiments from transient expressed protoplasts, the soluble fractions were prepared in IP buffer (15 mM Tris-HCl, pH 7.4, 150 mM NaCl, 0.5 mM EDTA, 0.1% Triton-X, 5% glycerol, and 1× Complete Protease Inhibitor Cocktail) and were then incubated with GFP–Trap agarose beads (ChromoTek) overnight at 4°C in a top-to-end rotator. After incubation, the beads were washed with co-immunoprecipitation buffer and then eluted by boiling in SDS sample buffer. Samples were separated by SDS-PAGE and analyzed by immunoblot using appropriate antibodies. Immunoblot analysis was performed as described previously (Shen et al., 2018).

### Drug Treatments

For drug-treatment experiments, ConcA (Sigma C9705) was added immediately after transfection of the protoplasts to obtain the final concentration of 200 nM ConcA, and confocal images were collected 8 h after incubation. BFA (Sigma B6542) and wortmannin (Sigma W1628) were prepared in DMSO and used at 10 μg/ml for 2 h and 16.5 μM for 1–2 h, respectively, in liquid medium. FM4-64 (Invitrogen T3166) uptake experiments were performed by incubation with FM4-64 (12 μM) for different time points before observation.

### Data Analysis

No statistical methods were used to predetermine samples or outcomes. Data were excluded when negative or positive controls were not working. Data are presented as mean values ± SD. Experiments were repeated independently at least three times. To determine if there is a statistically significant difference between samples of three or more independent groups, a complete randomized design was used at 5% significance level, and one-way analysis of variance (ANOVA) was performed using GraphPad Prism statistical analysis.

## Results

### Protoplast Isolation and PEG-Mediated Transient Expression in Moso Bamboo

**FIGURE 1:**
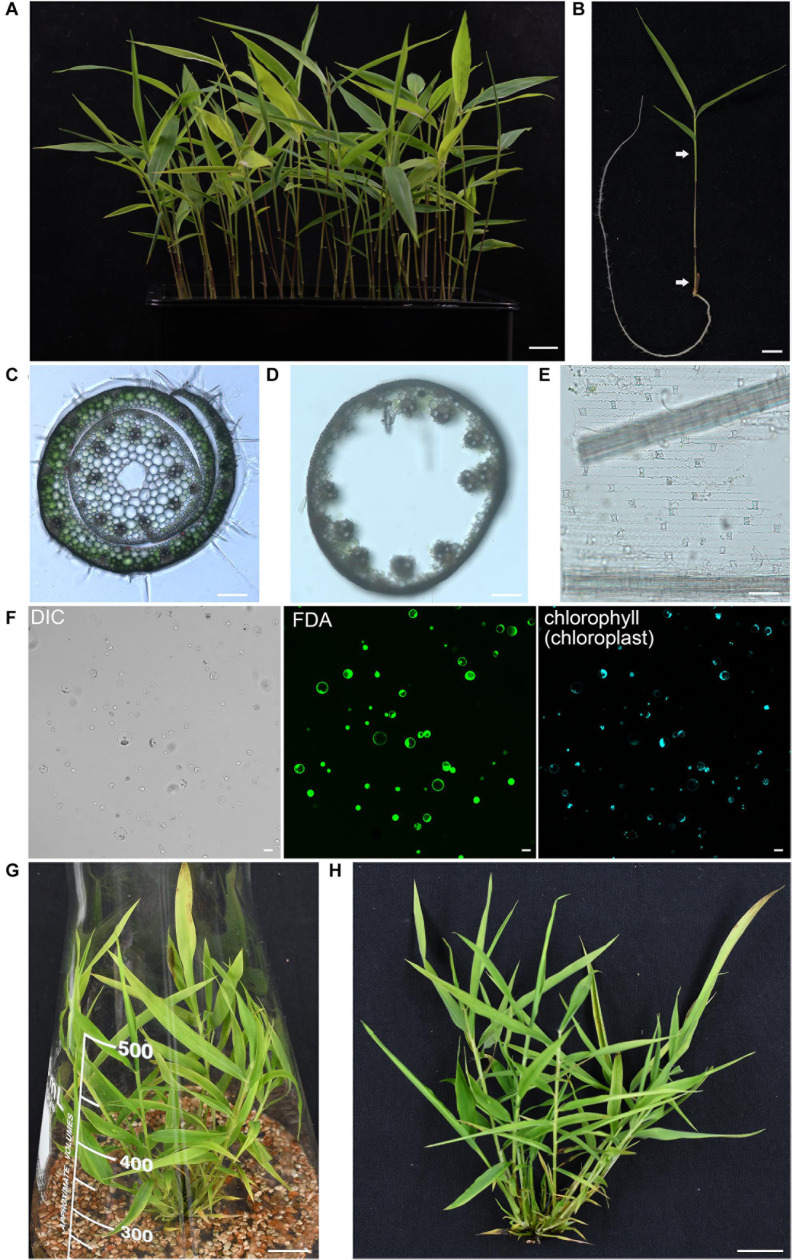
Isolation of protoplasts from moso bamboo seedling. **(A)** Morphology of 5-week-old greenhouse-grown green moso bamboo seedling used for protoplast isolation. Scale bar = 1.5 cm. **(B)** The culm and leaf sheath between the two arrows were cut in cross section and enzymatically digested for protoplast isolation. Scale bar = 1.5 cm. **(C)** A cross-sectional microscopic view of an intact culm with leaf sheath before enzyme digestion from a 5-week-old moso bamboo seedling. Scale bar = 10 μm. **(D,E)** Microscopic view of materials after enzyme digestion showing vascular bundles and epidermal cells in culm **(D)** or leaf sheath **(E)** remain, suggesting that protoplasts were mainly digested from cortical cells of culms and mesophyll cells of leaf sheaths. Scale bar = 10 μm. **(F)** FDA staining of isolated protoplasts showing a high level of vitality. Protoplasts were incubated with FDA for 2 min and then observed under confocal. DIC, differential interference contrast. Scale bar = 25 μm. **(G,H)** Morphology of 5-week-old micropropagated moso bamboo seedling **(G)**. The aerial part of seedlings **(H)** was used for protoplast isolation. Scale bar = 2 cm.

The enzyme combination of Cellulase R-10 and Macerozyme R-10 have been widely used for protoplasts isolation in *Arabidopsis thaliana* (Yoo et al., 2007), *Populus tremula* (Guo et al., 2012), *Oryza sativa* (Zhang Y. et al., 2011), *Zea mays* (Sheen, 2001), and *Triticum aestivum* (Jia et al., 2016). However, due to the different compositions of cell walls, the digestion enzymes for different plant materials should be optimized. In addition, mannitol, an osmoticum to facilitate cell wall digestion and the protoplasts to counteract turgor pressure, also need to be adjusted to optimum level (Yoo et al., 2007; Zhang Y. et al., 2011; Shen et al., 2014b). To obtain a high yield of protoplasts, we prepared multiple enzyme solutions according to previous published formula from different species (Supplementary Table 1) and then compared the digestion efficiencies on the fresh aerial part of seedlings from 5-week-old moso bamboo seedlings grown hydroponically in a growth chamber. We found that it is difficult to efficiently isolate protoplasts from leave blades, probably due to the wax and cuticle layer of epidermal surface in leaves (Wu et al., 2009). Thus, we used the fresh aerial part of seedlings containing culms and leaf sheaths as starting materials for protoplast isolation (Figures 1A,B). Culms and leaf sheaths were cut into strips and then incubated within various enzyme solutions for 4 h, after which the number of intact and independent protoplasts were counted under a microscope. Our optimized digestion condition included 0.4 M mannitol, 20 mM MES, pH 5.7, 20 mM KCl, 0.1% (w/v) bovine serum albumin, 10 mM CaCl_2_, 6% (w/v) Cellulase R-10, and 1.6% (w/v) Macerozyme R-10, which gave a protoplast yield of 8.0 × 10^6^/g FW. In our microscopic observation of digested tissues, we observed that the protoplasts were mostly released from cortical cells of culms and mesophyll cells of leaf sheaths, but most vascular bundles and epidermal cells remain (Figures 1C–E). A large proportion of the protoplasts imaged were spherical, indicating they remained intact after the isolation process (Figure 1F, left panel). The viability of the isolated protoplasts was 93.4 ± 2.4% as determined by fluorescein diacetate (FDA) staining (Figure 1F, middle panel), and most of the protoplasts contained chloroplasts (Figure 1F, right panel). There was a yield of approximately 2 × 10^7^ cells from 30 seedlings (5-week-old) digested for 4 h in the enzyme solution, which was sufficient for >20 transfection experiments (5 × 10^5^ protoplasts per transfection). In conclusion, the method described here enabled isolation of high quality yield of protoplasts from moso bamboo green seedlings. As an alternative material source for protoplast isolation, we also carried out protoplast isolation from micro-propagated moso bamboo seedlings (Figure 1G). We pooled together aerial tissues from 5- to 7-week-old seedlings grown in culture medium (Figure 1H). Similar protoplast yield and quality were achieved with a yield of 1.6 × 10^6^ protoplasts/g FW in the above optimized digestion condition. Thus, we used the protoplasts isolated from seedlings grown hydroponically in a growth chamber for the following experiments.

**FIGURE 2:**
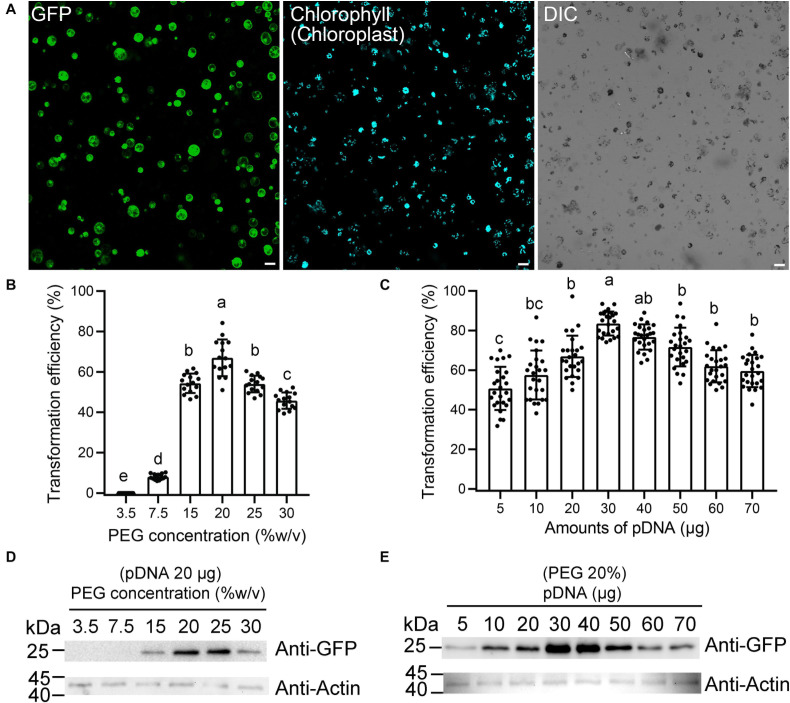
Optimization of moso bamboo protoplast transient expression system. **(A)** Representative images of the type of data acquired for this assay (a), showing green fluorescent protein (GFP) (green) and chlorophyll autofluorescence (cyan). DIC, differential interference contrast. Scale bar = 50 μm. **(B–E)** Assessment of protoplast transformation efficiency under varying final concentrations of polyethylene glycol (PEG) **(B,D)** or amounts of plasmid DNA (pDNA) **(C,E)**. The ratio of protoplast transformation **(B)** and Western blot **(D)** with constant amount of free GFP plasmids (20 μg) at varying concentrations of PEG. The ratio of protoplast transformation **(C)** and Western blot **(E)** at varying amounts of free GFP plasmid DNA (pDNA) under constant final concentration of PEG (20%). Protein extracts were prepared from protoplasts after transformation and analyzed by Western blot using anti-GFP and anti-actin antibodies. Actin was used as protein loading control. The ratio of protoplast transformation was calculated from at least 15 confocal images with three biological replicates (protoplasts isolated from three independent batches). Values and error bars are mean ± standard deviation (SD). For the different treatments, lowercase letters represent significant differences between sample means (*p* < 0.05).

The isolated protoplasts were then subjected to PEG-mediated gene transfection. According to previous reports, the transformation efficiency of large-sized plasmids was usually very low (Miao and Jiang, 2007; Hong et al., 2012). Therefore, to improve the transformation efficiency, the small-sized pBI221 vector (5,667 bp), by replacing the GUS coding sequence by functional fluorescence proteins (FPs), were constructed in this study. To resolve the key factors that may affect the transformation efficiency, protoplasts were transformed with an enhanced GFP expression plasmid, which shows fluorescence signal in the cytosol under confocal laser scanning microscopy (CLSM). The representative confocal image for the protoplast transfection under this system is shown in Figure 2A.

Next, we attempted to improve the protoplast transformation efficiency by optimizing different parameters, including plasmid concentration, PEG concentration, and transfection time. The experiments were carried out in a sequence as presented below. After each optimization step, the optimized condition was incorporated into the next set of optimization experiments. According to the rice protoplast transformation, we established a 220-μl transformation system, with 5 × 10^5^ protoplasts and 20 μg of plasmid DNA, to test the effects of the PEG concentration on the transfection efficiency. We found that the efficiency was the greatest (66.9 ± 9.1%) at 20% (w/v) PEG 4000 (Figure 2B). The transformation efficiency also reached to 54.0 ± 4.1% when 25% (w/v) PEG 4000 was used, but many protoplast fragments appeared under the microscope. Therefore, a final concentration of 20% PEG (w/v) was chosen as the optimum concentration, and then the variable amount of plasmid DNA was tested for transfection efficiency (Figure 2C). As expected, we observed an obvious increase in transfection efficiency when the amount of plasmid DNA was increased from 5 to 30 μg. The transfection efficiency peaked (83.6 ± 6.1%) with 30 μg of plasmid DNA, which demonstrated that a higher amount of plasmid DNA up to a certain threshold would significantly increase transfection efficiency. Finally, we used the final concentration of 20% PEG (w/v) with 20 μg of plasmids as a condition to test the effect of different transfection times on transformation efficiency. The transfection efficiency was highest (66.5 ± 5.8%) after a 15-min infection, while the efficiency was 37.8 ± 3.6% and 57.6 ± 6.2%, respectively, after 5- and 20-min infection. The expression levels of free GFP in this assay were also confirmed by Western blotting to test the effect of plasmid amount and PEG concentration. After a 12-h culture of transfection, the total protein extracted from equal amounts of cells was subjected to Western blotting using anti-GFP antibody. The 28-kDa bands corresponding to the GFP protein were detected. The Western blotting data demonstrated that 30 μg of pDNA or 20% (w/v) PEG was indeed positively correlated with the maximum GFP transfection efficiencies, respectively (Figures 2D,E). Collectively, our transformation conditions described in this study represent a high-efficiency protoplast transient gene expression system with approximately 83.6 ± 6.1% for single GFP transfection and 53.9 ± 5.2% for GFP and mRFP coexpression.

### Protein Subcellular Localization in Moso Bamboo Protoplasts

**FIGURE 3:**
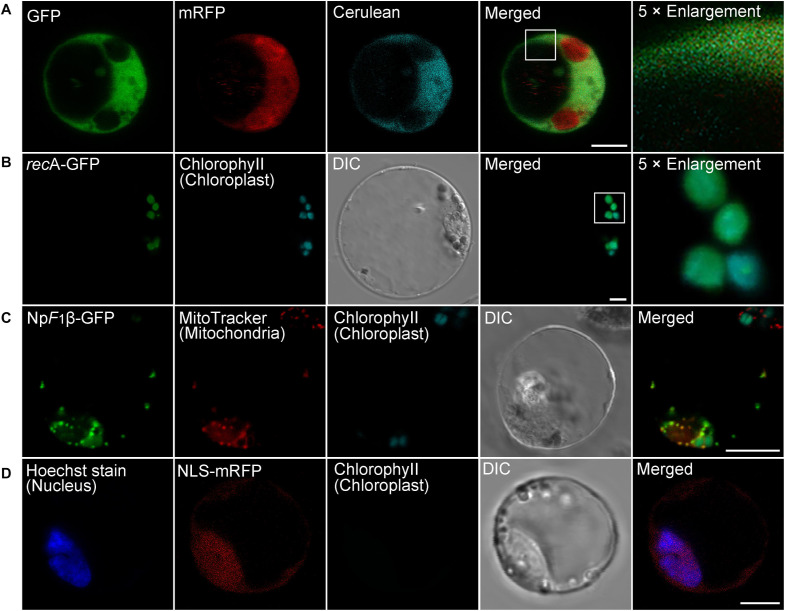
Subcellular localization of various organelle markers in moso bamboo protoplasts. **(A)** Fluorescent patterns of GFP (green), monomeric red fluorescent protein (mRFP) (red), and cerulean (cyan) in cotransformed protoplasts. **(B)** The chloroplast stroma marker *rec*A-GFP was transformed into protoplasts. The GFP signal (green) completely colocalized with chlorophyll autofluorescence (cyan). **(C)** The mitochondria matrix marker Np*F*_1_β–GFP was transformed into protoplasts, and transformed protoplasts were subsequently stained with MitoTracker for 5 min. The GFP signal (green) completely colocalized with the mitochondria signal (red). **(D)** The nucleus marker NLS–mRFP was transformed into protoplasts, and transformed protoplasts were subsequently stained with nuclear Hoechst for 5 min. The mRFP signal (red) colocalized with the nuclear Hoechst signal (blue). DIC, differential interference contrast. Scale bar = 5 μm.

Since many proteins are finally transported into particular intracellular compartments to play their specific functions, it is essential for us to know the subcellular localization of proteins of interest to determine their functions and interaction networks (Rojo and Denecke, 2008). To test the utility of the transient expression system for protein subcellular localization analysis, we first analyzed the localization of well-identified fluorescent fusion markers in moso bamboo protoplasts, including the *Arabidopsis* chloroplast stroma marker *rec*A–GFP (Köhler et al., 1997), the tobacco mitochondria matrix marker Np*F*_1_β-GFP–GFP (Duby et al., 2001), as well as the tobacco nucleus marker NLS–GFP (Grebenok et al., 1997). The expressed free cerulean, GFP, and mRFP without any targeting signal showed cytosolic fluorescent signals in protoplasts (Figure 3A), whereas in *rec*A-GFP transformed protoplasts, the green GFP signal colocalized with the red chlorophyll autofluorescence in the chloroplast (Figure 3B). Np*F*_1_β–GFP revealed fluorescence punctae throughout the cytoplasm, which colocalized with the MitoTracker dye (Figure 3C), indicating their mitochondria localization. Moreover, the nucleus marker NLS–mRFP colocalized specifically with the nuclear Hoechst (Figure 3D). Taken together, these colocalization results indicate that fusion proteins behaving as specific targeting sequences could locate to their correct cellular destination in our protoplast transient expression system. Next, we applied the organelle markers from the *Arabidopsis* endomembrane system, which has been developed in *Arabidopsis* protoplasts (Shen et al., 2013b; Zhu et al., 2020). However, we achieved low fluorescence signal or limited successful transformations of organelle markers tagged with mRFP in moso bamboo protoplasts, and in some cases, the fluorescence marker proteins (such as ER-localized mRFP–HDEL, *cis*-Golgi-localized mRFP–EMP12, or TGN/PM-localized SCAMP1–YFP) were not recruited to the supposed organelles. It is possible that ectopic expression of the gene of interest in plant cells usually ceases due to posttranscriptional gene silencing (Lu et al., 2017). Moreover, when we searched the moso bamboo homologs of *Arabidopsis* K/HDEL receptor ERD2 and p24δ subfamily proteins, which are involved in retrograde transport of K/HDEL ligand (Montesinos et al., 2014), it was interesting to find that the expression levels of these genes were very low, or even could not be detected, in different tissue samples (Supplementary Table 2). Similarly, the homologs of *Arabidopsis* putative EMP12 transport regulator (β-COP and ε-COP; Woo et al., 2015) were also expressed at low levels (Supplementary Table 2). Therefore, our preliminary gene expression analysis supports previous claims that ectopic expression of proteins may not faithfully reflect the native subcellular localization in different plant species because of the non-conservation of sorting machineries in the heterologous expression system (Marion et al., 2008; Collings, 2013). Thus, it is necessary to identify endogenous proteins, which has high expression level and correct targeting, as organelle markers in moso bamboo protoplasts.

### Endogenous Organelle Markers in the Endomembrane System of Moso Bamboo Protoplasts

Although multicolored fluorescent protein-based organelles targeting plasmids for transient expression or stable transgenic plants have been developed in *Arabidopsis* and other plants as *in vivo* organelle markers for subcellular colocalization studies (Tanz et al., 2013; Wu et al., 2016), no subcellular organelle markers have been reported for the bamboo species. All organelle markers reported here were generated with three different FPs (CFP/Cerulean, GFP, or mRFP) in pBI221 vector, to allow flexible combinations for subcellular localization analysis in moso bamboo protoplast transient expression. Table 1 gives an overview of the organelle marker set in the moso bamboo endomembrane system, containing the endoplasmic reticulum (ER), the Golgi apparatus, the *trans*-Golgi network or early endosome (TGN/EE), the prevacuolar compartment/multivesicular body or late endosome (PVC/MVB/LE), vacuole/tonoplast, and plasma membrane (PM). The subcellular localizations of the generated fluorescent markers were confirmed by the colocalization analysis with *Arabidopsis* organelle markers in both *Arabidopsis* and moso bamboo protoplasts. The labeled organelles in moso bamboo protoplasts displayed characteristic morphologies consistent with those previously reported in *Arabidopsis* or tobacco protoplasts (Wang et al., 2010; Denecke et al., 2012; Shen et al., 2013b).

**TABLE 1:**
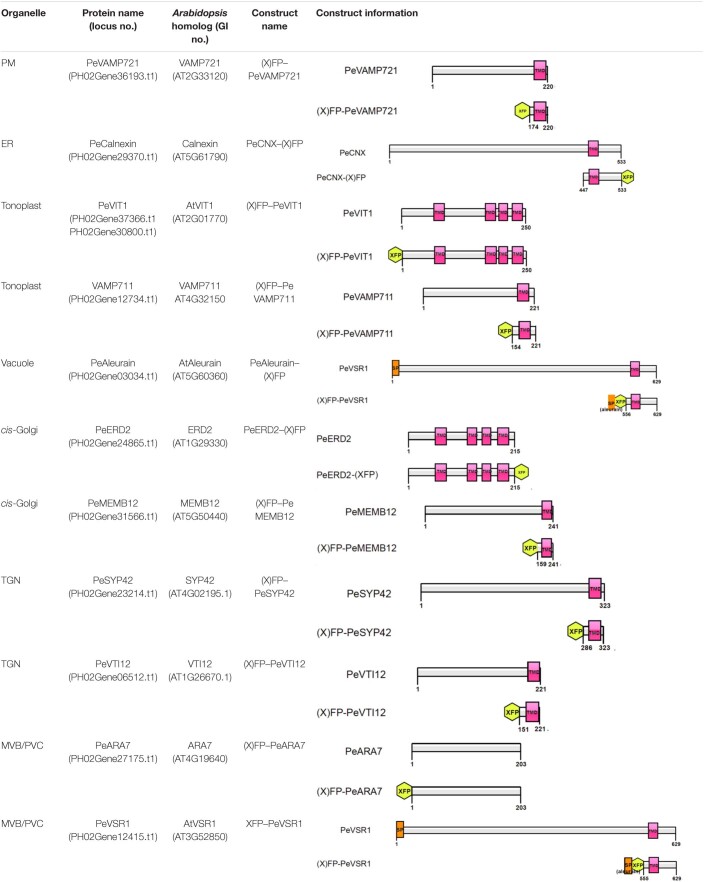
Construct information of the organelle markers in moso bamboo protoplasts.

**FIGURE 4:**
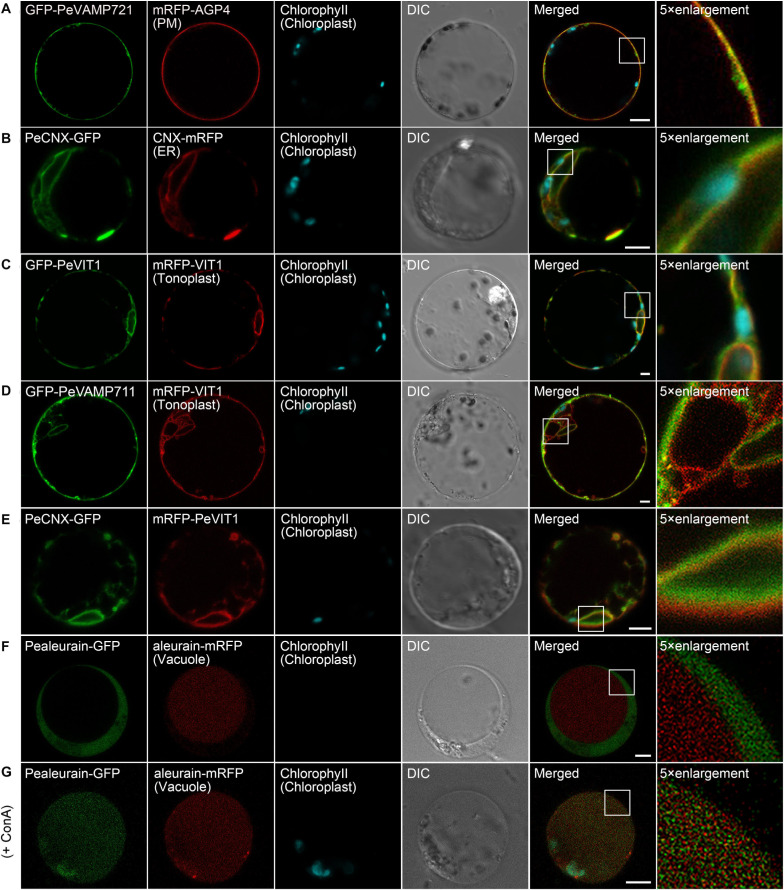
Subcellular localization of endogenous organelle markers on plasma membrane (PM), endoplasmic reticulum (ER), and vacuole of moso bamboo. The constructs were transiently expressed in moso bamboo protoplasts and visualized by confocal laser scanning microscopy (CLSM). **(A)** Colocalization of GFP–PeVAMP721 with *Arabidopsis* PM marker protein mRFP–AGP4. **(B)** Colocalization of PeCNX–GFP with *Arabidopsis* ER marker CNX–mRFP. **(C)** Colocalization of GFP–PeVIT1 with *Arabidopsis* tonoplast marker mRFP–VIT1. **(D)** Colocalization of GFP–PeVAMP711 with *Arabidopsis* tonoplast marker mRFP–VIT1. **(E)** Co-expression of moso bamboo ER marker protein PeCNX–GFP with tonoplast marker mRFP–PeVIT1. Note the separation of the green and red signals, suggesting the two organelle-specific markers. **(F)** Coexpression of PeAleurain–GFP with *Arabidopsis* lytic vacuole marker aleurain–mRFP. Note that the GFP signal in the vacuole lumen is quenched. **(G)** The PeAleurain–GFP shows fluorescence signals and colocalized with aleurain–mRFP in the vacuole lumen after ConcA treatment (200 nM). Moso bamboo protoplasts coexpressing PeAleurain–GFP with aleurain–mRFP were treated with ConcA immediately after transformation, followed by incubation for 8 h before confocal imaging. DIC, differential interference contrast. Scale bar = 5 μm.

The PM is the outer membrane of a cell that surrounds the cytoplasm. Hence, proteins that localize to the PM display uniform labeling along the surface of the cell. The soluble *N*-ethyl-maleimide-sensitive factor attachment protein receptor (SNARE) proteins VAMP721 and VAMP722, which are known to be responsible for protein secretion and extracellular defense, both have PM localization besides TGN under confocal observation (Uemrua et al., 2004; Zhang L. et al., 2011). Here, we applied protein sequence-based homology search and identified putative PeVAMP721 in moso bamboo. Under confocal microscopy, the GFP–PeVAMP721 colocalized with the mRFP-fused GPI–anchor arabinogalactan protein 4 (AGP4), mRFP–AGP4, an *Arabidopsis* PM marker in both moso bamboo and *Arabidopsis* protoplasts (Figure 4A and Supplementary Figure 1A). Therefore, we have confirmed the subcellular localization of FP-tagged PeVAMP721 as moso bamboo PM markers.

The ER forms as an interconnected system with flattened membrane sacks and tubules throughout the entire cytosol. It is continuously connected to the outer membrane of the nuclear envelope. The residence of the chaperone calnexin (CNX) in the ER is due to its transmembrane domain (TMD) and cytosolic tail (CT) (da Silva et al., 2005). Thus, the FP fusion of CNX–TMD/CT was created as an ER marker for live-cell imaging analysis in both *Arabidopsis* and tobacco protoplasts. In this study, we fused the FPs with the TMD/CT targeting sequence of the putative CNX protein (PeCNX–FPs) as potential ER markers in moso bamboo. The PeCNX-GFP exhibited prominent fluorescence around the nucleus, forming extensive networks throughout the cytoplasm (Figure 4B). The fluorescent signal of PeCNX–GFP colocalized with the *Arabidopsis* ER marker CNX–mRFP in both moso bamboo and *Arabidopsis* protoplasts (Figure 4B and Supplementary Figure 1B), suggesting that the PeCNX–FPs could be used as ER markers.

The tonoplast is the delimiting membrane around the vacuole; membrane proteins reaching the tonoplast require multiple signals involving ER export and tonoplast targeting sequence. In a previous work, the *Arabidopsis* multiple transmembrane domain protein vacuolar ion transporter1 (VIT1) has been reported to localize at the tonoplast in *Arabidopsis* (Kim et al., 2006; Wang et al., 2014). Moreover, SNARE proteins, including the VAMP711, have also been demonstrated to locate to the tonoplast (Sato et al., 1997). In this study, the fluorescence signal of GFP–PeVIT1 was fully expanded throughout the cell revealing the large size of the central vacuole and was colocalized with the *Arabidopsis* tonoplast marker mRFP–VIT1 under CLSM analysis in both moso bamboo and *Arabidopsis* protoplasts (Figure 4C and Supplementary Figure 1C). Similarly, the fluorescence signals of moso bamboo SNARE protein GFP–PeVAMP711 also colocalized with the *Arabidopsis* tonoplast marker mRFP–VIT1 (Figure 4D and Supplementary Figure 1D) Thus, we here provide FPs–PeVIT1 and FPs–PeVAMP711 as markers for the tonoplast in moso bamboo protoplasts. Because both ER and tonoplast display tubular network in moso bamboo protoplasts, we attempted to distinguish the two organelles by our generated markers under CLSM observation. Indeed, when transient coexpression of PeCNX–GFP and mRFP–PeVIT1 in moso bamboo protoplasts, the fluorescence signal of PeCNX–GFP is in close proximity, but apparently separated, to the tonoplast marker mRFP–PeVIT1 (Figure 4E), suggesting the identity of the two organelle markers.

In the secretory pathway, soluble proteins reach the vacuolar lumen because they contain certain vacuolar sorting determinants (VSDs) that can be recognized by the vacuolar sorting receptors (VSRs) (Neuhaus and Rogers, 1998). Up to now, using the targeting sequence of the vacuolar soluble proteins, several FP fusion reporters have been used in the protoplast transient expression as vacuolar markers, including aleurain–FPs (containing aleurain N-terminus VSD) (Fluckiger et al., 2003). In this study, we homology searched the putative moso bamboo aleurain protein PeAleurain, and the N-terminus sequence containing the potential VSD was N-terminally fused to the FPs. CLSM analysis revealed that the PeAleurain–GFP presented some punctate structures in the cytoplasm but without signal in the vacuole, while the aleurain–mRFP marker had a strong red signal in the vacuole (Figure 4F). It is reported that soluble GFP in the lumen of vacuoles often lack fluorescence signals because of the low pH of the vacuole (Tamura et al., 2003). To further confirm that the abundance of protons inside the vacuole quenched PeAleurain–GFP fluorescence, the protoplasts were treated with concanamycin A (ConcA), which can inhibit vacuolar-type H^+^-ATPase (V-ATPase) activity and raise the internal vacuole pH (Tamura et al., 2003; Gaxiola et al., 2007; Krebs et al., 2010). Indeed, after treatment with ConcA (200 nM) in transfected protoplasts, the PeAleurain–GFP fluorescence in the vacuole was restored and showed colocalization with aleurain–mRFP in CLSM analysis (Figure 4G and Supplementary Figure 1E).

**FIGURE 5:**
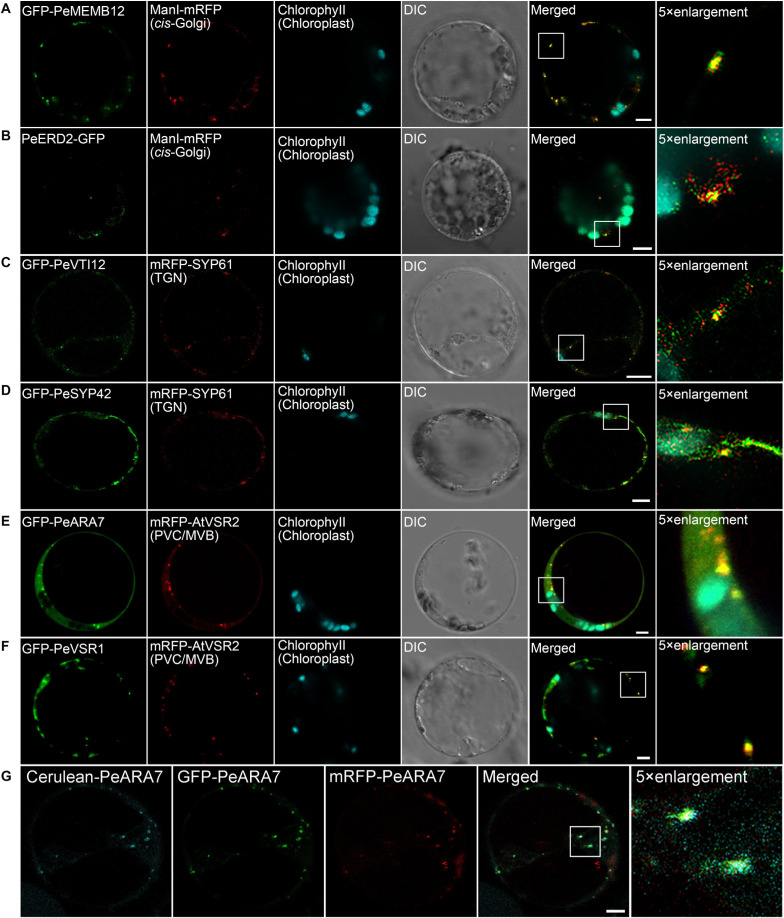
Subcellular localization of endogenous organelle markers in endosomal compartments of moso bamboo. **(A,B)** Colocalization of GFP–PeMEMB12 **(A)** or PeERD2–GFP **(B)** with *Arabidopsis cis*-Golgi marker ManI–mRFP. **(C,D)** Colocalization of GFP–PeVTI12 **(C)** or GFP–PeSYP42 **(D)** with *Arabidopsis* TGN marker mRFP–AtSYP61. **(E,F)** Colocalization of GFP–PeARA7 **(E)** or GFP–PeVSR1 **(F)** with *Arabidopsis* PVC/MVB marker mRFP–AtVSR2. **(G)** Examples of colocalization signals of the PVC/MVB compartment marker PeARA7 tagged with cerulean (cyan), GFP (green), or mRFP (red) transiently expressed in moso bamboo protoplasts. Note that all three fluorophores allow clear spectral separation, based on their excitation/emission characteristics. DIC, differential interference contrast. Scale bar = 5 μm.

Next, we try to identify endogenous proteins as endosomal compartments in moso bamboo. The Golgi apparatus consists of several stacked cisternae near the outer edges of the ER and organized into three biochemically distinct subcompartments: the *cis*-Golgi, *medial*-Golgi, and *trans*-Golgi (Brandizzi et al., 2002). In previous studies, *Arabidopsis* Golgi-localized SNARE protein MEMB12 fused to FP has been used as a *cis*-Golgi-specific marker (Uemura et al., 2004). Moreover, the KDEL receptor ERD2 has been found mainly localized to both the punctate Golgi structures and the ER network in protoplasts (Pastor-Cantizano et al., 2018). Here, we applied protein sequence-based homology search and identified putative MEMB12 and ERD2 homologs in moso bamboo. The sequence behaving as the TMD and CT regions of PeMEMB12 was fused to the C-terminus of FPs for localization analysis. Under CLSM analysis, GFP–PeMEMB12 showed punctate fluorescence within the cytoplasm, which colocalized with the *Arabidopsis cis*-Golgi marker ManI–mRFP in both moso bamboo and *Arabidopsis* protoplasts (Figure 5A and Supplementary Figure 2A). Similarly, PeERD2-GFP also showed punctate pattern and colocalized with ManI–mRFP (Figure 5B and Supplementary Figure 2B). These data suggested that both PeMEMB12 and PeERD2 located at the *cis*-Golgi and can be used as *cis*-Golgi markers in moso bamboo protoplasts.

The TGN is a specialized organelle with a tubular reticulum on the *trans* side of the Golgi stack. The TGN not only functions as an early endosome (EE), which receives internalized materials from the plasma membrane, but also provides a final sorting station for Golgi-derived cargoes for delivery to the vacuoles. The TGN/EE-localized SNARE protein, SYNTAXIN OF PLANTS 61 (SYP61) has been reported to mediate vacuolar trafficking with the SYP4 group (SYP41, SYP42, and SYP43), VPS TEN INTERACTING 12 (VTI12) in addition to the SM (Sec1/Munc18) protein VACUOLAR PROTEIN SORTING 45 (VPS45) (Zouhar et al., 2009). At present, SYP41, SYP42, SYP61, and VTI12 have been used as TGN markers in *Arabidopsis* cells (Bassham et al., 2000; Sanderfoot et al., 2001). In this study, the TMD and CT regions of the putative VTI12 protein in moso bamboo were fused with the FPs and coexpressed with *Arabidopsis* TGN marker SYP61–mRFP. GFP–PeVTI12 was shown as a typical TGN localization pattern and colocalized with *Arabidopsis* mRFP–SYP61 under CLSM (Figure 5C and Supplementary Figure 2C). Similarly, the punctate structures of GFP–PeSYP42, and GFP fusion of the TMD and CT regions of putative PeSYP42 were also colocalized with the *Arabidopsis* TGN marker mRFP–SYP61 (Figure 5D and Supplementary Figure 2D). These data suggest that the PeVTI12 and PeSYP42 both can be used as TGN markers in moso bamboo protoplasts.

The plant prevacuolar compartment (PVC) or multivesicular body (MVB) is an organelle that not only participates in the secretory pathway that mediates protein trafficking to vacuoles but also serves in the endocytic pathway as a late endosome (LE) in plants (Hu et al., 2020a). Because VSRs are mainly concentrated on PVC/MVBs in sorting acid hydrolases to vacuoles (Sanderfoot et al., 1998), VSR reporter proteins, containing the TMD and CT regions of VSRs, were first used as an organelle marker to define the PVC (Tse et al., 2004). In addition, Rab5, which is critical for PVC/MVB maturation, lytic vacuole biogenesis, and in regulating vacuolar protein trafficking in plants, has also been localized at PVC/MVB (Sohn et al., 2003; Geldner, 2004; Jia et al., 2013; Cui et al., 2014). Thus, the two homologs of Rab5, RHA1 and ARA7, are also used as PVC/MVB markers in *Arabidopsis* (Sohn et al., 2003; Lee et al., 2004). In our study, homology search returned the putative PeVSR1 and PeARA7 in moso bamboo. When the GFP–PeARA7 and *Arabidopsis* PVC/MVB marker mRFP–AtVSR2 were transiently coexpressed in moso bamboo protoplasts, they largely colocalized to the punctate organelles (Figure 5E and Supplementary Figure 2E). Moreover, the GFP–PeVSR1 was also localized in a typical punctate PVC pattern, which colocalized with the mRFP–AtVSR2 (Figure 5F and Supplementary Figure 2F). These data suggest that the PVC/MVB localization of PeARA7 and PeVSR1 can be used as PVC/MVBs markers in moso bamboo protoplasts.

The above characterized protein sequences were finally fused with mRFP or CFP/cerulean to allow flexible combinations for subcellular localization analysis in moso bamboo protoplasts transient expression. The colocalization of different fluorescent tag-fused markers (Figure 5G and Supplementary Figures 3, 4) confirmed the viability of the multicolored organelle markers for subcellular localization analysis in protoplasts of moso bamboo.

### Validation of Endosome Markers by Endocytic Dye and Pharmaceutical Treatment

**FIGURE 6:**
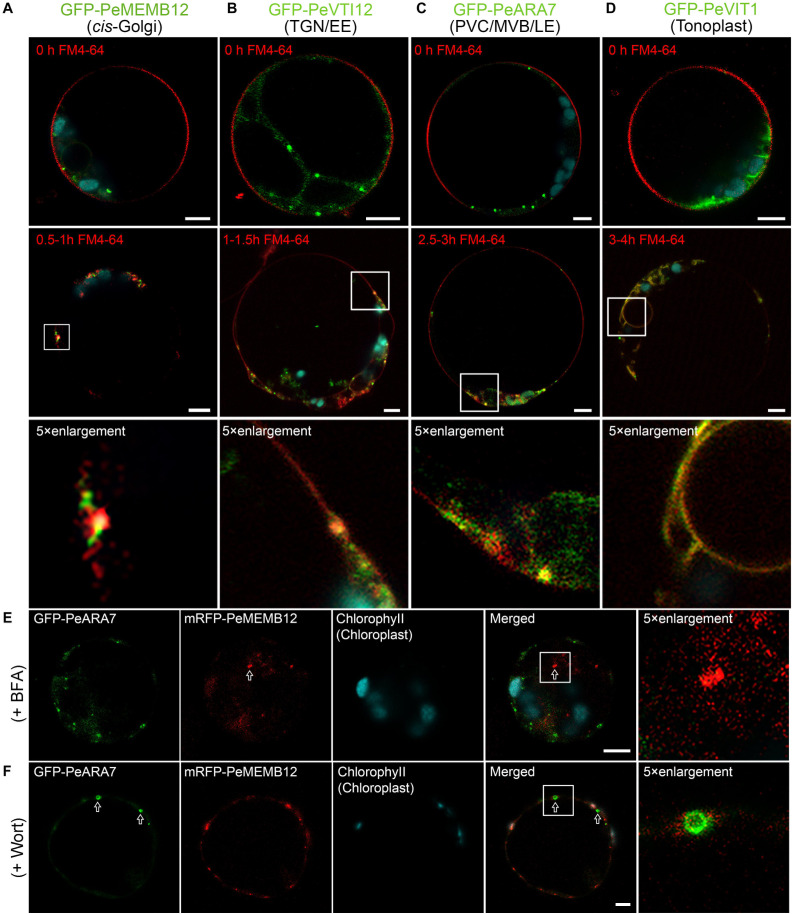
Functional mapping of endosomal compartment markers by combinatorial colocalization. **(A–D)** Time course of FM4-64 uptake in moso bamboo protoplasts expressing different endosomal markers. Colocalization analysis of GFP-labeled compartments (green) with the endocytic tracer FM4-64 (red) after 0.5–1 h (**A**, middle panel), 1–1.5 h (**B**, middle panel), 2.5–3 h (**C**, middle panel), or 3–4 h (D, middle panel) of uptake. Some compartments can be clearly defined as colocalizing or non-colocalizing, whereas many show partial colocalization (low panel). Note the time-dependent colocalization of early endosome (EE) TGN or late endosome (LE) prevacuolar compartment/multivesicular body (PVC/MVB) marker with FM4-64. **(E)** Subcellular localization analysis of PVC/MVB localized GFP–PeARA7 with *cis*-Golgi localized mRFP–MEMB12 after brefeldin A (BFA) treatment. The arrow indicates the mRFP–MEMB12 forms large aggregation, while GFP–PeARA7 does not form aggregates. **(F)** Subcellular localization analysis of GFP–PeARA7 with mRFP–MEMB12 after wortmannin (Wort) treatment. Arrows indicate that GFP–PeARA7 forms ring-like structures, while mRFP–MEMB12 shows no response. Scale bar = 5 μm.

The endosomal membrane system is highly complex and dynamic, and the most ill-defined aspect of plant cell compartmentation. To evaluate how well endosomal compartments were represented in our marker set, we employed the fluorescent styryl dye FM4-64 to investigate the localization of the established moso bamboo endosome markers. Generally, the internalized endosomal marker FM4-64 from the PM reaches the early endosome (EE) or TGN before reaching the late endosome (LE) or PVC/MVB and subsequently the lytic vacuole (LV) (Emans et al., 2002; Lam et al., 2007). Thus, we performed the time course of FM4-64 dye arrival into GFP-labeled endosome markers after short (30 min) to long (4 h) periods of uptake in moso bamboo protoplasts. As shown in Figure 6, the red FM4-64 dye rapidly stained the PM and was separated from the individual endosome markers. After a 0.5- to 1.5-h uptake, the Golgi marker GFP–PeMEMB12 showed little colocalization with the internalized FM4-64 (Figure 6A, middle and bottom panel), whereas the TGN marker GFP–PeVTI12 showed significant colocalization with FM4-64 after a 1- to 1.5-h uptake (Figure 6B, middle and bottom panel). Similarly, the internalized FM4-64 did not colocalize with the PVC marker GFP–PeARA7 during the first 2 h of uptake, but showed typical colocalization after 2.5–3 h of uptake (Figure 6C, middle and bottom panel). Finally, the internalized FM4-64 reached the tonoplast and colocalized with the GFP–PeVIT1 after 3–4 h of uptake (Figure 6D, middle and bottom panel). Therefore, we conclude that the internalized FM4-64 reached the GFP–PeVTI12-labeled EE/TGN compartments before the GFP–PeARA7-labeled LE/PVC/MVB compartments.

Visible changes in fluorescent protein-labeled organelles in response to chemical drug treatments such as wortmannin or brefeldin A (BFA) have also been widely used to manipulate and define plant endosomes (Geldner et al., 2003). We further confirmed the subcellular localization of the endosome markers in moso bamboo by pharmaceutical treatment. The BFA is an inhibitor of a subset of Sec7 domain-containing ADP ribosylation factor (ARF) guanine nucleotide exchange factors (ARF-GEF) (Jackson and Casanova, 2000). At low concentrations (5–10 μg/ml), BFA could cause both the Golgi apparatus and TGN to form visible aggregations, called BFA bodies or BFA compartments (Lam et al., 2009). Wortmannin is an inhibitor of phosphatidylinositol-3 kinase (PI-3 kinase, Vps34p in yeast) (Schu et al., 1993; Corvera et al., 1999). Upon wortmannin treatment, PVCs/MVBs become vacuolated as ring-like structures because of the homotypical PVC fusion (Tse et al., 2004). In our experiments, when protoplasts coexpressing the TGN marker GFP–PeVTI12 and mRFP–SYP61, aggregated fluorescent compartments were observed after BFA treatment (Supplementary Figure 5A). By contrast, when the same concentration of BFA was used to treat the protoplasts coexpressing the PVC marker GFP–PeARA7 and mRFP–PeMEMB12, no enlarged BFA compartment was detected on the PVC marker GFP–PeARA7 (Figure 6E), whereas in protoplasts treated with wortmannin, the GFP–PeARA7 and the PVC marker mRFP–AtVSR2 both colocalized on the membrane of the ring-like structures (Supplementary Figure 5B). Likewise, the mRFP–PeMEMB12 had no response to the wortmannin treatment, while the coexpressed GFP–PeARA7 showed enlarged vacuolated PVC (Figure 6F). Therefore, by tracing the endocytic pathway and pharmaceutical treatment on organelle marker-labeled endosomes, we have confirmed the subcellular localization of our new generated endosome-targeted proteins.

In conclusion, based on the targeting sequences of endogenous proteins in the endomembrane system of moso bamboo, we here generated a set of multicolored organelle markers with different FPs that provides a valuable resource for determining the subcellular localization of previously uncharacterized proteins and can also be used for visualizing organelle dynamics in living moso bamboo cells.

### A Demonstration of the Transient Gene Expression System to Evaluate the Subcellular Localization and Transport Dynamic of Uncharacterized Proteins

**FIGURE 7:**
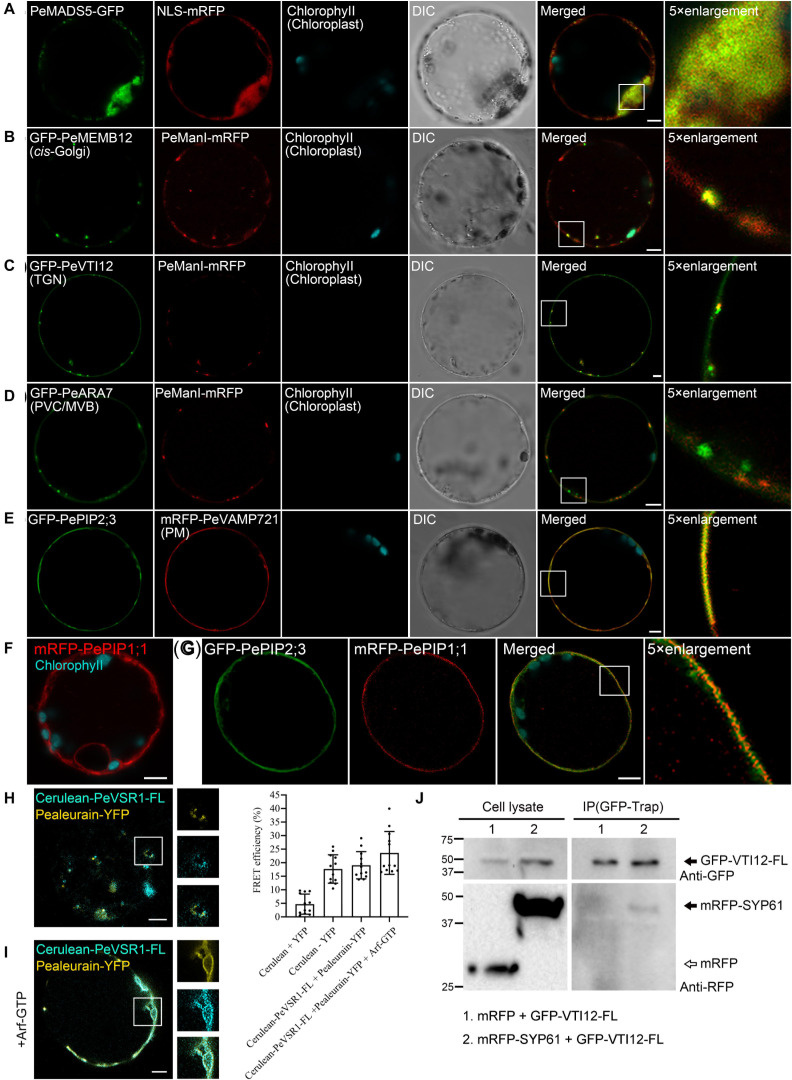
Subcellular localization and protein–protein interaction analysis of uncharacterized proteins in moso bamboo protoplasts. **(A)** Colocalization of PeMADS5–GFP with the nucleus marker NLS–mRFP. **(B–D)** Subcellular localization of moso bamboo PeManI fused to mRFP in protoplasts. Coexpression of PeManI–mRFP with *cis*-Golgi marker GFP–PeMEMB12 **(B)**, TGN marker GFP–PeVTI12 **(C)**, and PVC/MVB marker GFP–PeARA7 **(D)**. Note that PeManI–mRFP colocalized with the *cis*-Golgi marker, associated with the *trans*-Golgi network (TGN) marker, but separated from the PVC/MVB marker. **(E–G)** Subcellular localization of PePIPs fused to GFP in moso bamboo protoplasts. Colocalization of GFP–PePIP2;3 with the PM marker mRFP–VAMP721 **(E)**. mRFP–PePIP1;1 fluorescence **(F)**. **(G)** Coexpression of GFP–PePIP2;3 and mRFP–PePIP1;1. Note the subcellular localization of mRFP–PePIP1;1 is relocalized from ER to plasma membrane by coexpression of GFP–PePIP2;3. **(H,I)** Acceptor photobleaching-fluorescence resonance energy transfer (FRET-AB) analysis between vacuolar sorting receptor (PeVSR1) and vacuolar cargo PeAleurain. Cerulean–PeVSR1–FL and PeAleurain–YFP were coexpressed in moso bamboo protoplasts followed by confocal imaging (left panel) and FRET-AB quantifications (right panel). The method to quantify FRET efficiency is described in the *Materials and Methods* section. For each group, at least 10 individual protoplasts were used for FRET efficiency quantification and statistical analysis. **(J)** Co-immunoprecipitation (co-IP) analysis between GFP-tagged full-length VTI12 (GFP–VTI12–FL) and mRFP-tagged SYP61 (mRFP–SYP61). Proteins were isolated from moso bamboo protoplasts coexpressing mRFP (lane 1) or mRFP–SYP61 (lane 2) with GFP–VTI12–FL as indicated, followed by immunoprecipitation (IP) using GFP–Trap agarose beads and subsequent immunoblot analysis on eluted proteins using anti-GFP or anti-RFP antibodies. mRFP was used as negative control. Arrow indicates VTI12. White and black arrowheads indicate mRFP and mRFP–SYP61, respectively. DIC, differential interference contrast. Scale bar = 5 μm.

To test the utility of the multicolored organelle marker in a transient expression system for colocalization analysis, we next characterized the subcellular localizations of several uncharacterized proteins, which may play potential functions in moso bamboo flowering and shoot fast growth. MADS-box genes encode a family of transcription factors that play specialized roles in developmental processes in flowering plants ranging from root to flower and fruit development. In previous studies, expression analysis of the bamboo MADS-box genes in floral organs suggested that PeMADS5 acts as a potential floral activator and may be involved in bamboo flowering (Zhang et al., 2018). As a transcription factor, the PeMADS5 is supposed to be localized at the nucleus. Indeed, the PeMADS5–GFP, a full-length PeMADS5 fusion with GFP, was localized in the nucleus and colocalized with the nucleus marker NLS–mRFP in moso bamboo protoplast (Figure 7A). Thus, the subcellular localization results further support its function as a transcription factor.

Next, we attempt to study the subcellular localization of moso bamboo class I α-mannosidases and the plasma membrane intrinsic protein (PIP) aquaporins, which are highly expressed in fast growth stage of bamboo shoot (Zhao et al., 2014; Ma et al., 2018). In *Arabidopsis*, class I α-mannosidases play functions in early *N*-glycan-processing reactions, which are essential for cell wall architecture and plant root growth (Liebminger et al., 2009). To determine the subcellular localization of the putative class I α-mannosidase (PH02Gene02226.t1) in moso bamboo, the PeManI–mRFP was constructed, and the fusion proteins appeared as fluorescence mainly in small mobile vesicles under CLSM analysis (Figure 7B and Supplementary Movie 1), which was significantly colocalized with the characterized *cis*-Golgi marker GFP–PeMEMB12, but not the TGN marker GFP–PeVTI12 or PVC marker GFP–PeARA7, respectively (Figures 7B–D). These data are consistent with the research result from Golgi localization of the soybean enzyme (Nebenführ et al., 1999; Saint-Jore-Dupas et al., 2006) and in agreement with the proposed function of the enzymes in the Golgi apparatus. In plants, PIP aquaporins are main water channels at the plant plasma membrane, which are crucial molecular players involved in numerous essential physiological processes, including long-distance water flux, cell water homeostasis, and cell elongation (Chaumont and Tyerman, 2014). Similar with other plants, the PIP subfamily in moso bamboo is subdivided into two groups, namely, PIP1s and PIP2s. Here, we want to identify the subcellular localization of the two putative PIP1;1 (PH02Gene43726.t1) and PIP2;3 (PH02Gene40446.t1). When expressed in moso bamboo protoplasts, PePIP2;3-GFP are found in the plasma membrane (PM) and colocalized with the PM marker VAMP721–mRFP (Figure 7E), whereas PePIP1;1-GFP was retained in the ER (Figure 7F). However, when coexpressed with PePIP2;3-mRFP, PePIP1;1-GFP was relocalized to the PM (Figure 7G). Similar results have also been reported in ZmPIP1s and ZmPIP2s, and the relocalization results are possibly due to the formation of hetero-oligomers between ZmPIP1s and ZmPIP2s (Zelazny et al., 2007). Collectively, these data confirm that the transient transformation system and the organelle markers generated here is ideally suited for subcellular localization studies.

### Protein–Protein Interaction Analysis of Uncharacterized Proteins in Protoplasts

To identify and visualize protein–protein interactions *in vivo* or *in vitro* is helpful to understand the protein interaction network and thereby their molecular functions. In this study, we attempted to apply the moso bamboo protoplast system in studying protein–protein interaction by acceptor photobleaching fluorescence resonance energy transfer (FRET-AB) analysis. The main function of VSRs is sorting soluble vacuolar cargoes to vacuoles in plants (Kirsch et al., 1994; Paris et al., 1997; Shen et al., 2013a, 2014a). A subcellular localization study indicated that VSRs are concentrated on PVC/MVBs, although it is suggested that the VSRs can start sorting cargo proteins in the ER (Niemes et al., 2010a, b; Kunzl et al., 2016). We next attempted to confirm the interaction of PeVSR1 and PeAleurain by FRET analysis between cerulean–PeVSR1 and PeAleurain–YFP (Figure 7H). Free cerulean coexpressing with free EYFP was used as a negative control, while the cerulean–EYFP fusion constructs were expressed as a positive control for the FRET analysis. As shown in Figure 7H, coexpression of the cerulean-tagged full-length PeVSR1 (cerulean–PeVSR1–FL) and PeAleurain–YFP fusion constructs resulted in a clear colocalization signal in punctae but showed weak interactions. Recent reports demonstrated that the VSR–cargo interaction can occur at the ER lumen, then we tried to coexpress the *Arabidopsis* Arf–GTP mutant, which blocks the secretion pathway of the VSR–cargo from the ER to the Golgi. After coexpressing Arf–GTP, cerulean–PeVSR1 and PeAleurain–YFP colocalized in the ER lumen and showed strong interactions (Figure 7I).

Next, we used the moso bamboo protoplast transient expression system to investigate protein–protein interaction for co-immunoprecipitation (co-IP) assay. VTI12 (Qb-SNARE), SYP61 (Qc-SNARE), and YKT6 (R-SNARE) have been reported to form a SNARE complex with the SYP4 proteins (SYP41, SYP42, and SYP43) at the TGN (Zouhar et al., 2009; Kim and Brandizzi, 2012). Thus, the moso bamboo homologs of VTI12 and SYP61 were used as a pair to test the validity of our system for co-IP experiments. The full-length PeVTI12 and PeSYP61 were fused with FPs, respectively. GFP–PeVTI12–FL were transiently expressed together with mRFP–PeSYP61 or mRFP in protoplasts, followed by protein extraction for co-IP using GFP–Trap and subsequent Western blotting after co-IP in immunoprecipitates using anti-GFP or anti-RFP antibodies. The negative control mRFP did not show interaction with GFP–PeVTI12 because no protein band was detected by anti-RFP in the immunoprecipitates, whereas mRFP–PeSYP61 was found to interact with GFP–PeVTI12 (Figure 7J). Therefore, the moso bamboo protoplast transient expression system can perform co-IP of two putative proteins with tag antibodies, which overcomes the potential concerns of specific antibody preparation and transgenic plant generation. Thus, these data demonstrated that the moso bamboo protoplast transient expression system can be applied for both the *in vitro* and *in vivo* protein–protein interaction.

## Discussion

Transient gene expression in plant protoplasts is a key technique in plant molecular cell biology research, since a high expression level of gene expression could be achieved rapidly to study gene function, protein subcellular localization, and protein–protein interactions (Denecke et al., 2012). A highly efficient gene transient expression system in moso bamboo is highly demanded in the post-genomic era for gene function studies due to the lack of transgenic system and long life cycle of this species. Here, a highly efficient moso bamboo protoplast-based transient expression system was developed, and the suitability of this system for studying gene functions *in vivo* was validated by showing the correct subcellular localization of introduced proteins and protein–protein interactions. Moreover, we characterized a set of multicolored endogenous organelle markers for subcellular localization studies of previously uncharacterized proteins. With these new resources described here, it is feasible to characterize gene subcellular localization and possible molecular functions *in vivo* rapidly and in high throughput in moso bamboo.

### Critical Factors in the Transient Gene Expression of Moso Bamboo Protoplasts

Usually, mesophyll protoplasts from leaf are a good choice for gene transient transformation (Sheen, 2001; Yoo et al., 2007). However, unlike the *Arabidopsis* and tobacco leaves, moso bamboo leaves are covered with an epicuticular wax layer and has a high silicon content (Lux et al., 2003), which blocks the enzyme solution infiltration into the leaves during protoplast isolation (Wu et al., 2009). Consequently, the yield of protoplasts from moso bamboo leaves is very low. In our experiments, we found that the culms and leaf sheaths with reduced silicon deposition from young seedlings are ideal materials for protoplast isolation, which is similar with that in rice using young leaf sheaths as starting materials (Shen et al., 2014b; Page et al., 2019).

Previous protoplast isolation and transformation protocols have emphasized the need for vacuum infiltration of plasmolyzed tissue with enzyme solution prior to the long-time incubation in order to obtain high protoplast yields (Yoo et al., 2007). Indeed, we have also applied vacuum infiltration treatment to ensure excellent yields of protoplasts. In our studies, we have also optimized the transformation condition in plasmid amount, PEG-4000 concentration, and transformation time in order to achieve high transformation efficiency. Consequently, the single transformation efficiency of pBI221–GFP was found to be up to 83.6%, while the co-transformation efficiency of GFP and mRFP was found to be approximately 53.9%, which is significantly higher than previous reports on protoplasts of other bamboo species (Lin et al., 2018; Ye et al., 2020). In conclusion, the optimal plant starting materials associated with highly efficient protoplast isolation, the optimal enzyme formula, the yield of protoplasts, the pDNA and protoplast amount, the PEG concentration and transformation time, and the estimate of transformation efficiency had not been described previously in bamboo species. In this work, we have reported these parameters, which are essential for the efficient application of this protoplast transient expression system to test the large numbers of expression cassettes rapidly.

### Organelle Markers in the Moso Bamboo Endomembrane System

It should be noted that studies for bamboo protein subcellular localization and protein–protein interaction have been reported only in heterologous expression. Usually, bamboo proteins are fluorescence tagged and expressed in rice protoplasts, onion epidermal cells, or tobacco leaves (Ge et al., 2017; Liu et al., 2018; Xiao et al., 2018). However, different expression patterns may arise from heterologous systems (Marion et al., 2008), and we have also found that several fluorescence-tagged proteins were functional in the *Arabidopsis* transient expression system but were not expressed or did not show similar subcellular distribution in moso bamboo protoplasts. Thus, homologous systems with high efficiency are much more highly preferred to address protein function, as protein endosomal transport and interactions may be regulated by endogenous scaffold or partner proteins (Smyczynski et al., 2006). Therefore, the moso bamboo protoplast transient expression system we have developed here can eliminate potential problems.

In the present work, we generated a set of different FP-tagged organelle markers using moso bamboo endogenous proteins or its targeting sequences (Table 1), which are relevant for protein localization in moso bamboo protoplasts. We found consistent expression patterns of these organelle markers in both *Arabidopsis* and moso bamboo protoplasts. In addition, most of the organelle markers were generated by the fusion of FPs with short targeting sequences from moso bamboo proteins in order to reduce the possibility of introducing any new protein activity that might alter cellular trafficking behavior. The organelle targeting sequences are based on the homolog sequences from *Arabidopsis* (Nelson et al., 2007; Shen et al., 2013b; Zhu et al., 2020). We have also confirmed the localization of our moso bamboo organelle markers with the corresponding mRFP-tagged *Arabidopsis* organelle markers in both moso bamboo and *Arabidopsis* protoplasts (Figures 4, 5 and Supplementary Figures 1, 2). Moreover, the confocal data of endosome markers by the endocytic FM4-64 dye staining and responses to pharmaceutical treatments further demonstrated that each endosome marker was targeted to the correct destination and thus provided further support for the usefulness of our moso bamboo organelle markers in subcellular localization studies and organelle dynamic tracking (Figure 6 and Supplementary Figure 5). Additionally, researchers can use these organelle markers for generating transgenic lines with stable localization markers in moso bamboo when the transgenic system is established.

### The Moso Bamboo Protoplast Transient Expression Allows Determination of Protein–Protein Interactions

Traditionally, co-IP is a technology used for protein–protein interaction identification. However, co-IP could not be widely performed due to the difficulties in raising specific antibodies and plant transgenesis (Miernyk and Thelen, 2008). Our studies overcome these drawbacks by using moso bamboo protoplasts transiently expressing epitope-tagged proteins to perform co-IP experiments. The co-IP of the protein pair, GFP–VTI12-FL and mRFP–SYP61, has been successfully performed using GFP or RFP tag antibodies to confirm the interaction in transformed moso bamboo protoplasts (Figure 7J). In addition, this system can also be applied to investigate protein–protein interactions by FRET analysis of proteins localized at the same organelle (Figures 7H,I). Therefore, our studies provide highly reliable and convenient techniques in moso bamboo protoplasts for the determination of interactions of candidate proteins.

## Conclusion

Given the advantages of the moso bamboo transient expression system laid out above, in addition to the set of organelle markers generated here, this platform could be used to solve a wide range of research questions. Examples are responses to abiotic environmental stimuli required for flowering or bamboo shoot fast growth, as well as cell signaling pathways in bamboo plant defense against pathogen. In all of these cases, the cellular responses are the key points in understanding those processes. This protoplast transient expression system in moso bamboo could be used for large-scale, high-throughput co-localization analysis of proteins and protein–protein interactions (Sheen, 2001; Yoo et al., 2007). It could also be used in gene functional studies, for example, by comparing the relative expression level of different promoters and the rapid screening of target sequences of transcriptional factor after transient chromatin immunoprecipitation-sequencing (ChIP-seq) in protoplasts (Lee et al., 2017). Additionally, the transient expression system presented here could also be used to identify the efficiency of gRNAs in CRISPR/Cas9 genome editing (Lin et al., 2018; Ye et al., 2020) or even assess more recent advancements in CRISPR/Cas9 technology such as DNA methylation editing or base editing (Gallego-Bartolomé et al., 2018; Chen et al., 2019).

## Data Availability Statement

The original contributions presented in the study are included in the article/Supplementary Material, further inquiries can be directed to the corresponding author.

## Author Contributions

JS, MZ, SH, and XL designed the research. MZ, SH, FY, YW, and YG performed the experiments. JS, MZ, SH, YC, DH, and XL analyzed the data. JS and SH wrote the manuscript with comments from all authors.

## Conflict of Interest

The authors declare that the research was conducted in the absence of any commercial or financial relationships that could be construed as a potential conflict of interest. The reviewer CG declared a past co-authorship with several of the authors JS, DZ to the handling editor.

## Footnotes

1 www.arabidopsis.org

2 www.bamboogdb.org

3 http://www.cbs.dtu.dk/services/SignalP/

4 http://www.cbs.dtu.dk/services/tmhmm-2.0/

5 http://www.adobe.com/

## Supplementary Material

Supplementary Figure 1 Related to Figure 4. Subcellular localization of organelle markers from moso bamboo on PM, ER, and vacuole in *Arabidopsis* protoplasts. **(A)** Co-expression of GFP-PeVAMP721 with *Arabidopsis* PM marker protein mRFP-AGP4. **(B)** Co-expression of PeCNX-GFP with *Arabidopsis* ER marker CNX-mRFP. **(C)** Co-expression of GFP-PeVIT1 with *Arabidopsis* tonoplast marker mRFP-AtVIT1. **(D)** Co-expression of GFP-PeVAMP711 with *Arabidopsis* tonoplast marker mRFP-AtVIT1. **(E)** Co-expression of PeAleurain–GFP with *Arabidopsis* lytic vacuole marker aleurain-mRFP. Protoplasts were treated with ConcA (200 nM) immediately after transformation, followed by incubation for 8 h before confocal imaging. DIC, differential interference contrast. Scale bar = 5 μm.

Supplementary Figure 2 Related to Figure 5. Subcellular localization of organelle markers from moso bamboo on endosomal compartments in *Arabidopsis* protoplasts. **(A,B)** Co-expression of GFP-PeMEMB12 **(A)** or PeERD2-GFP **(B)** with *Arabidopsis cis*-Golgi marker ManI-mRFP. **(C,D)** Co-expression of GFP-PeVTI12 **(C)** or GFP-PeSYP42 **(D)** with *Arabidopsis* TGN marker mRFP-AtSYP61. **(E,F)** Co-expression of GFP-PeARA7 **(E)** or GFP-PeVSR1 **(F)** with *Arabidopsis* PVC/MVB marker mRFP-AtVSR2. DIC, differential interference contrast. Scale bar = 5 μm.

Supplementary Figure 3 Related to Figure 4. Colocalization analysis of different fluorescent tag fused markers on PM, ER, and vacuole in moso bamboo protoplasts. **(A)** Colocalization of GFP-PeVAMP721 with mRFP-PeVAMP721. **(B)** Colocalization of PeCNX-GFP with PeCNX-mRFP. **(C)** Colocalization of GFP-PeVIT1 with mRFP-PeVIT1. **(D)** Colocalization of GFP-PeVAMP711 with mRFP-PeVAMP711. **(E)** Colocalization of PeAleurain–GFP with aleurain-mRFP after ConcA (200 nM) treatment. DIC, differential interference contrast. Scale bar = 5 μm.

Supplementary Figure 4 Related to Figure 5. Colocalization analysis of different fluorescent tag fused markers on endosomal compartments in moso bamboo protoplasts. **(A)** Colocalization of GFP-PeMEMB12 or with mRFP-PeMEMB12. **(B)** Colocalization of PeERD2-GFP with PeERD2-mRFP. **(C)** Colocalization of GFP-PeVTI12 with mRFP-PeVTI12. **(D)** Colocalization of GFP-PeSYP42 with mRFP-PeSYP42. **(E)** Colocalization of GFP-PeARA7 with mRFP-PeARA7. **(F)** Colocalization of GFP-PeVSR1 with mRFP-PeVSR1. DIC, differential interference contrast. Scale bar = 5 μm.

Supplementary Figure 5 Related to Figure 6. Response of endosomal markers to pharmaceutical treatment in moso bamboo protoplasts. **(A)** Subcellular localization analysis of TGN localized GFP-PeVTI12 with *Arabidopsis* TGN marker mRFP-SYP61 after BFA treatment. **(B)** Subcellular localization analysis of GFP-PeARA7 with *Arabidopsis* PVC/MVB marker mRFP-AtVSR2 after wortmannin (Wort) treatment. Scale bar = 5 μm.

Supplementary Table 1 Combinations of digestion enzymes used.

Supplementary Table 2 Examples of Expression profiles of intracellular vesicle trafficking regulators in moso bamboo.

Supplementary Table 3 Detailed information of primer sequences for each construct.
