# Supplementary material for: Organelle Visualization With Multicolored Fluorescent Markers in Bamboo
Source: Front Plant Sci. 2021 Apr 15;12:658836. doi: 10.3389/fpls.2021.658836 (PMC8081836; doi:10.3389/fpls.2021.658836)
Supplement: Supplementary Figure 1 — Related to Figure 4. Subcellular localization of organelle markers from moso bamboo on PM, ER, and vacuole in Arabidopsis protoplasts. (A) Co-expression of GFP-PeVAMP721 with Arabidopsis PM marker protein mRFP-AGP4. (B) Co-expression of PeCNX-GFP with Arabidopsis ER marker CNX-mRFP. (C) Co-expression of GFP-PeVIT1 with Arabidopsis tonoplast marker mRFP-AtVIT1. (D) Co-expression of GFP-PeVAMP711 with Arabidopsis tonoplast marker mRFP-AtVIT1. (E) Co-expression of PeAleurain–GFP with Arabidopsis lytic vacuole marker aleurain-mRFP. Protoplasts were treated with ConcA (200 nM) immediately after transformation, followed by incubation for 8 h before confocal imaging. DIC, differential interference contrast. Scale bar = 5 μm. [file Data_Sheet_1.PDF]

SUPPLEMENTAL FIGURES

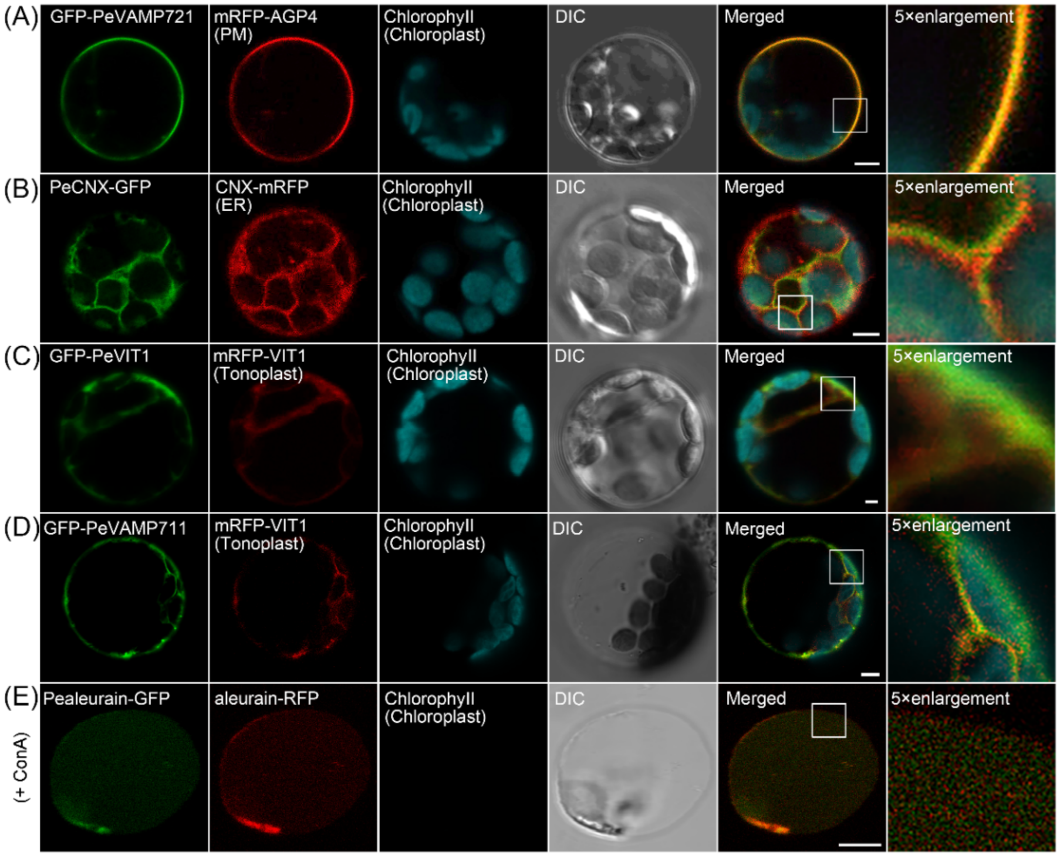

Supplementary Fig. 1, related to Fig. 4. Subcellular localization of organelle markers from moso bamboo on PM, ER, and vacuole in Arabidopsis protoplasts.

(A) Co-expression of GFP-PeVAMP721 with Arabidopsis PM marker protein mRFP-AGP4;

(B) Co-expression of PeCNX-GFP with Arabidopsis ER marker CNX-mRFP;

(C) Co-expression of GFP-PeVIT1 with Arabidopsis tonoplast marker mRFP-AtVIT1;

(D) Co-expression of GFP-PeVAMP711 with Arabidopsis tonoplast marker mRFP-AtVIT1;

(F) Co-expression of Pealeurain-GFP with Arabidopsis lytic vacuole marker aleurain-mRFP. Protoplasts were treated with ConcA (200 nM) immediately after transformation, followed by incubation for 8 h before confocal imaging.

DIC, differential interference contrast. Scale bar = 5 µm.

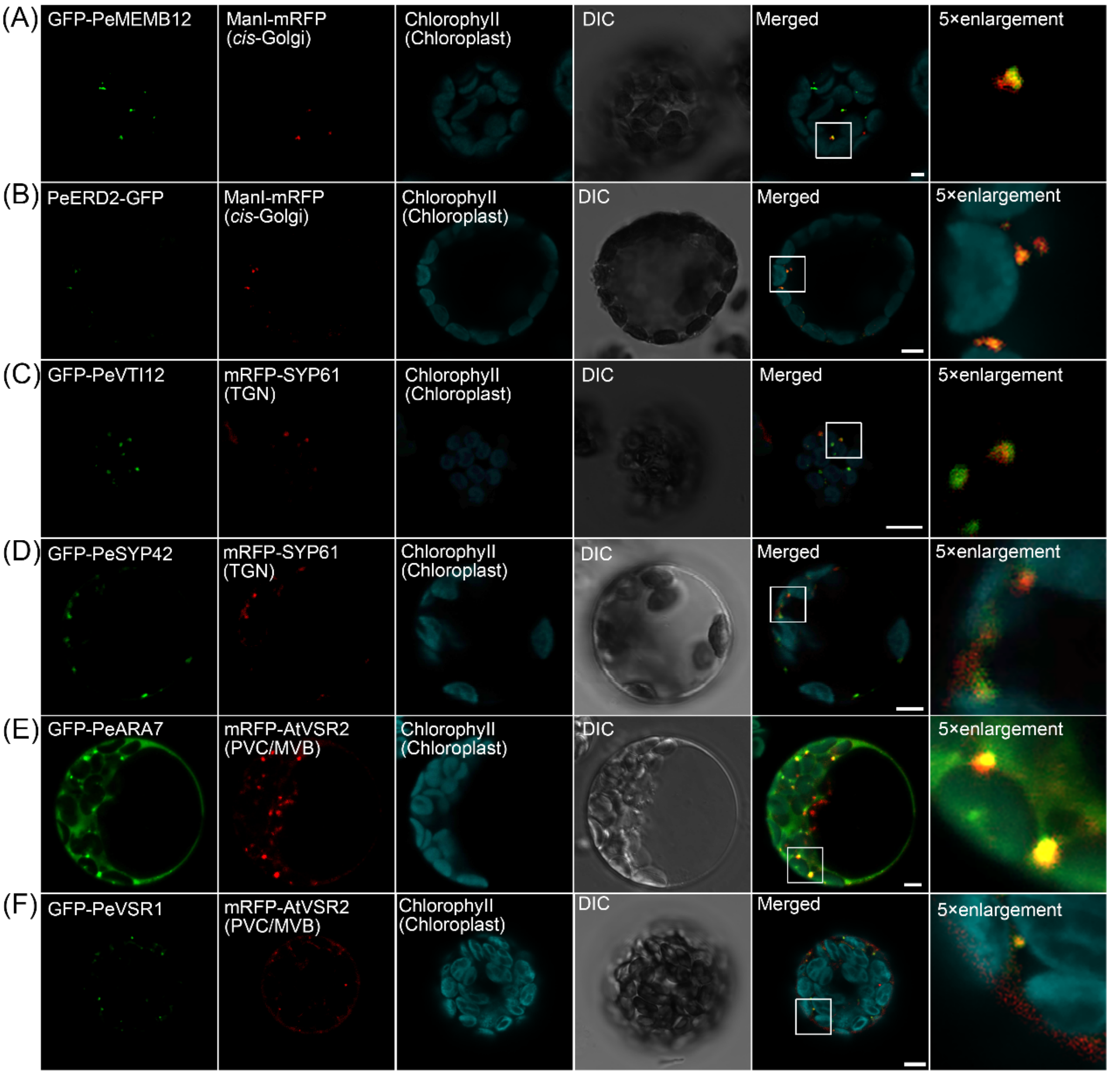

Supplementary Fig. 2, related to Fig. 5. Subcellular localization of organelle markers from moso bamboo on endosomal compartments in Arabidopsis protoplasts.

(A-B) Co-expression of GFP-PeMEMB12 (A) or PeERD2-GFP (B) with Arabidopsis *cis*-Golgi marker ManI-mRFP.

(C-D) Co-expression of GFP-PeVTI12 (C) or GFP-PeSYP42 (D) with Arabidopsis TGN marker mRFP-AtSYP61.

(E-F) Co-expression of GFP-PeARA7 (E) or GFP-PeVSR1 (F) with Arabidopsis PVC/MVB marker mRFP-AtVSR2.

DIC, differential interference contrast. Scale bar = 5 µm.

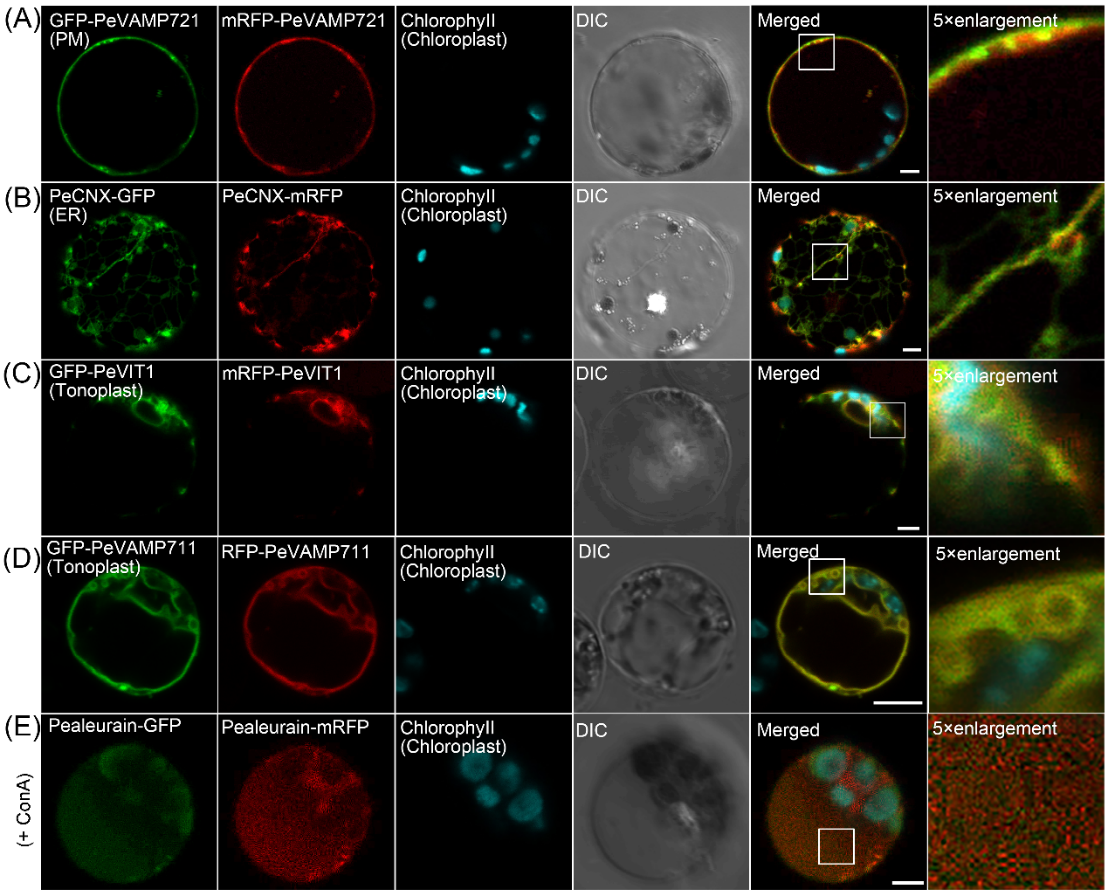

Supplementary Fig. 3, related to Fig. 4. Colocalization analysis of different fluorescent tag fused markers on PM, ER, and vacuole in moso bamboo protoplasts.

(A) Colocalization of GFP-PeVAMP721 with mRFP-PeVAMP721;

(B) Colocalization of PeCNX-GFP with PeCNX-mRFP;

(C) Colocalization of GFP-PeVIT1 with mRFP-PeVIT1;

(D) Colocalization of GFP-PeVAMP711 with mRFP-PeVAMP711;

(E) Colocalization of Pealeurain-GFP with aleurain-mRFP after ConcA (200 nM) treatment.

DIC, differential interference contrast. Scale bar = 5 μm.

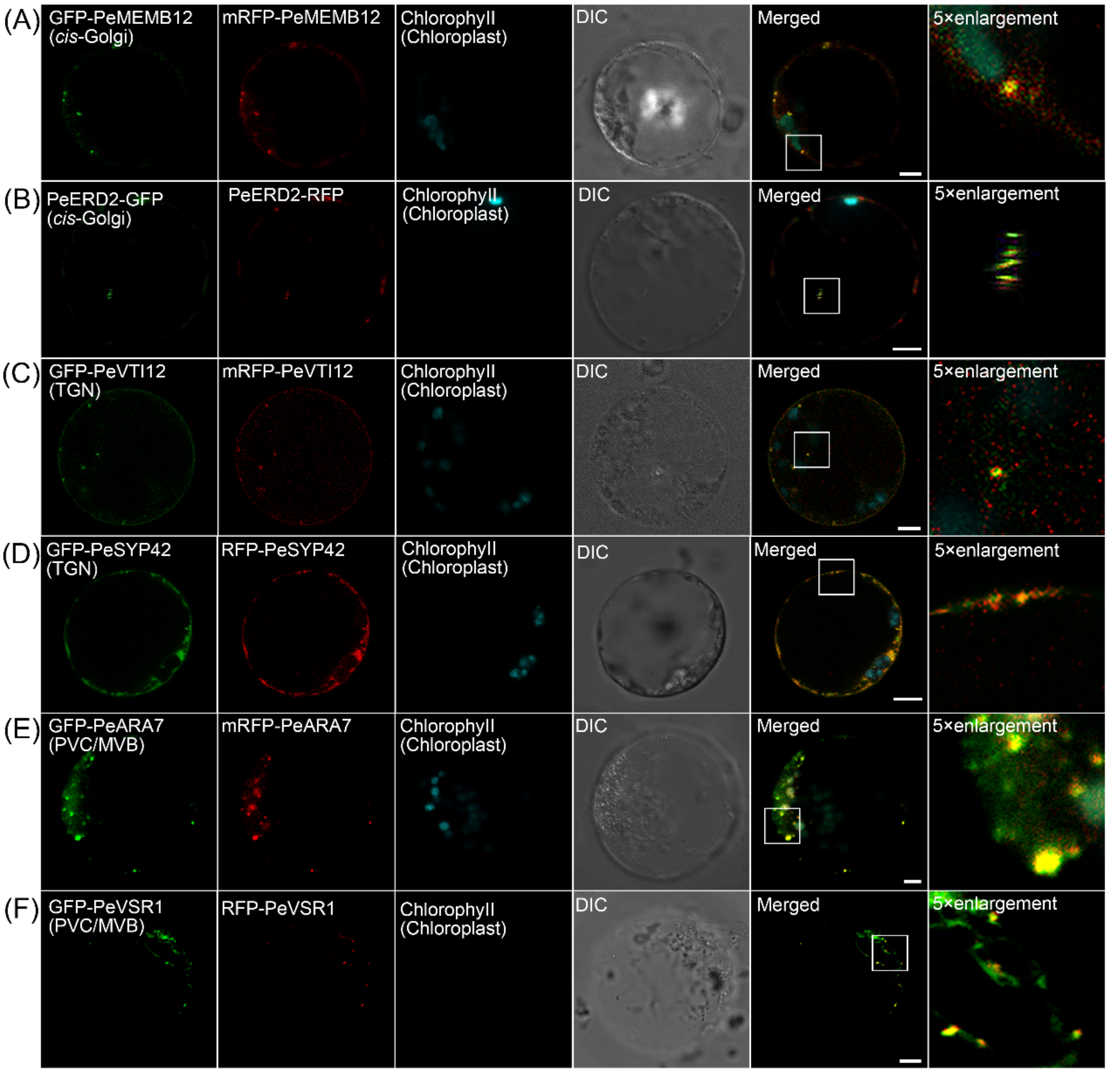

Supplementary Fig. 4, related to Fig. 5. Colocalization analysis of different fluorescent tag fused markers on endosomal compartments in moso bamboo protoplasts.

(A) Colocalization of GFP-PeMEMB12 or with mRFP-PeMEMB12;

(B) Colocalization of PeERD2-GFP with PeERD2-mRFP;

(C) Colocalization of GFP-PeVTI12 with mRFP-PeVTI12;

(D) Colocalization of GFP-PeSYP42 with mRFP-PeSYP42;

(E) Colocalization of GFP-PeARA7 with mRFP-PeARA7;

(F) Colocalization of GFP-PeVSR1 with mRFP-PeVSR1;

DIC, differential interference contrast. Scale bar = 5 µm.

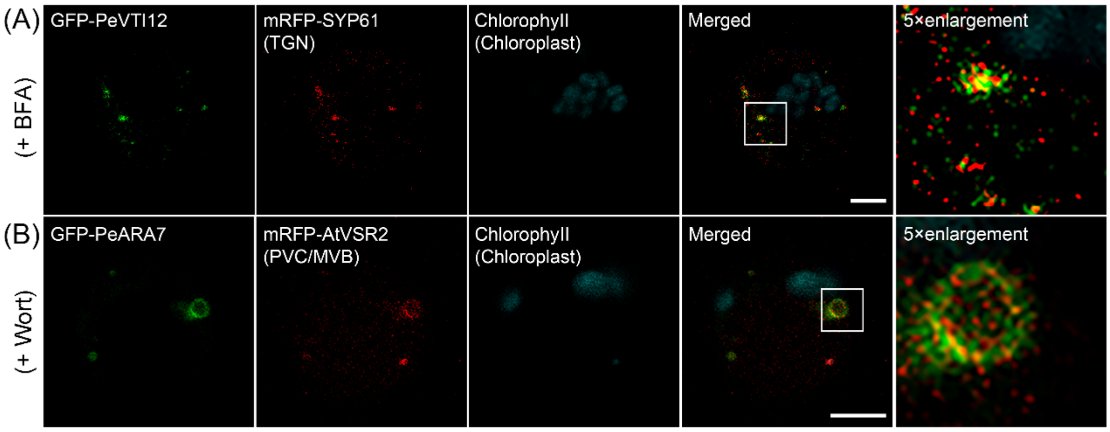

Supplementary Fig. 5, related to Fig. 6. Response of endosomal markers to pharmetical treatment in moso bamboo protoplasts.

(A) Subcellular localization analysis of TGN localized GFP-PeVTI12 with Arabidopsis TGN marker mRFP-SYP61 after BFA treatment.

(G) Subcellular localization analysis of GFP-PeARA7 with Arabidopsis PVC/MVB marker mRFP-AtVSR2 after wortmannin (Wort) treatment.

Scale bar = 5 µm.
