# Supplementary material for: Organelle Visualization With Multicolored Fluorescent Markers in Bamboo
Source: Front Plant Sci. 2021 Apr 15;12:658836. doi: 10.3389/fpls.2021.658836 (PMC8081836; doi:10.3389/fpls.2021.658836)
Supplement: Supplementary Table 1 — Combinations of digestion enzymes used. [file Table_1.DOCX]

**Supplementary Table 1. Combinations of digestion enzymes used.**

| **Enzyme Formula** | **Material & Protoplasts Isolation Efficiency** | **References** |
| --- | --- | --- |
| \| 1.5% (wt/vol) Cellulase R-10 \| \| --- \| \| 0.4% (wt/vol) Macerozyme R-10 \| \| 20 mM MES \| \| 0.4 M mannitol \| \| 20 mM KCl \| \| 10 mM CaCl_2_ \| \| 0.1% (wt/vol) BSA \| | 1 ×10^4^～2 ×10^5^ /g | (Shen et al., 2014) |
| \| 1.5% (w/v) Cellulase RS \| \| --- \| \| 0.75% (w/v) Macerozyme R-10 \| \| 10mM MES \| \| 0.5M mannitol \| \| 10 mM CaCl_2_ \| \| 0.1% (wt/vol) BSA \| | 1×10^4^～1×10^5^ /g | (Shen et al., 2014) |
| \| 1.5% (wt/vol) CellulaseR-10 \| \| --- \| \| 0.75% (wt/vol) MacerozymeR-10 \| \| \| 10 mM MES \| \| \| 0.6 M mannitol \| \| \| 1mM CaCl_2_ \| \| \| 0.1% (wt/vol) BSA \| \| | 1×10^4^～1×10^5^ /g | (Mazarei et al., 2008) |
| \| 3% (wt/vol) CellulaseR-10 \| \| --- \| \| 0.8% (wt/vol) MacerozymeR-10 \| \| \| 20 mM MES \| \| \| 0.4 M mannitol  20 mM KCl \| \| \| 10 mM CaCl_2_ \| \| \| 0.1% (wt/vol) BSA \| \| | 1×10^4^～1.7×10^5^ /g | - (Guo et al., 2012) |
| \| 3% (wt/vol) CellulaseR-10 \| \| --- \| \| 0.8% (wt/vol) MacerozymeR-10 \| \| \| 0.04% (w/v) Pectinase  20 mM MES \| \| \| 0.4 M mannitol  20 mM KCl \| \| \| 10 mM CaCl_2_ \| \| \| 0.1% (wt/vol) BSA \| \| | 1×10^4^～1×10^5^ /g | (Shen et al., 2017) |

**Supplementary References**

Guo, J., Morrell-Falvey, J.L., Labbé, J.L., Muchero, W., Kalluri, U.C., Tuskan, G.A. and Chen, J.G. (2012) Highly efficient isolation of Populus mesophyll protoplasts and its application in transient expression assays. *PLoS One* **7**, e44908.

Mazarei, M., Al-Ahmad, H., Rudis, M.R. and Stewart, C.N., Jr. (2008) Protoplast isolation and transient gene expression in switchgrass, Panicum virgatum L. *Biotechnol. J.* **3**, 354-359.

Shen, J., Fu, J., Ma, J., Wang, X., Gao, C., Zhuang, C., Wan, J. and Jiang, L. (2014) Isolation, Culture, and Transient Transformation of Plant Protoplasts. *Curr. Protoc. Cell Biol.* **63**, 2.8.1-2.8.17.

Shen, Y., Meng, D., McGrouther, K., Zhang, J. and Cheng, L. (2017) Efficient isolation of Magnolia protoplasts and the application to subcellular localization of MdeHSF1. *Plant Methods* **13**, 44.
