# Supplementary material for: Organelle Visualization With Multicolored Fluorescent Markers in Bamboo
Source: Front Plant Sci. 2021 Apr 15;12:658836. doi: 10.3389/fpls.2021.658836 (PMC8081836; doi:10.3389/fpls.2021.658836)
Supplement: Supplementary Table 2 — Examples of Expression profiles of intracellular vesicle trafficking regulators in moso bamboo. [file Table_2.DOCX]

**Supplementary Table 2. Examples of Expression profiles of intracellular vesicle trafficking regulators in moso bamboo.**

| ***Arabidopsis thaliana*** | ***Phyllostachys edulis*** | **Expression profile**^*^ |
| --- | --- | --- |
| AT5G09810  (actin7) | PH02Gene38192.t1 | 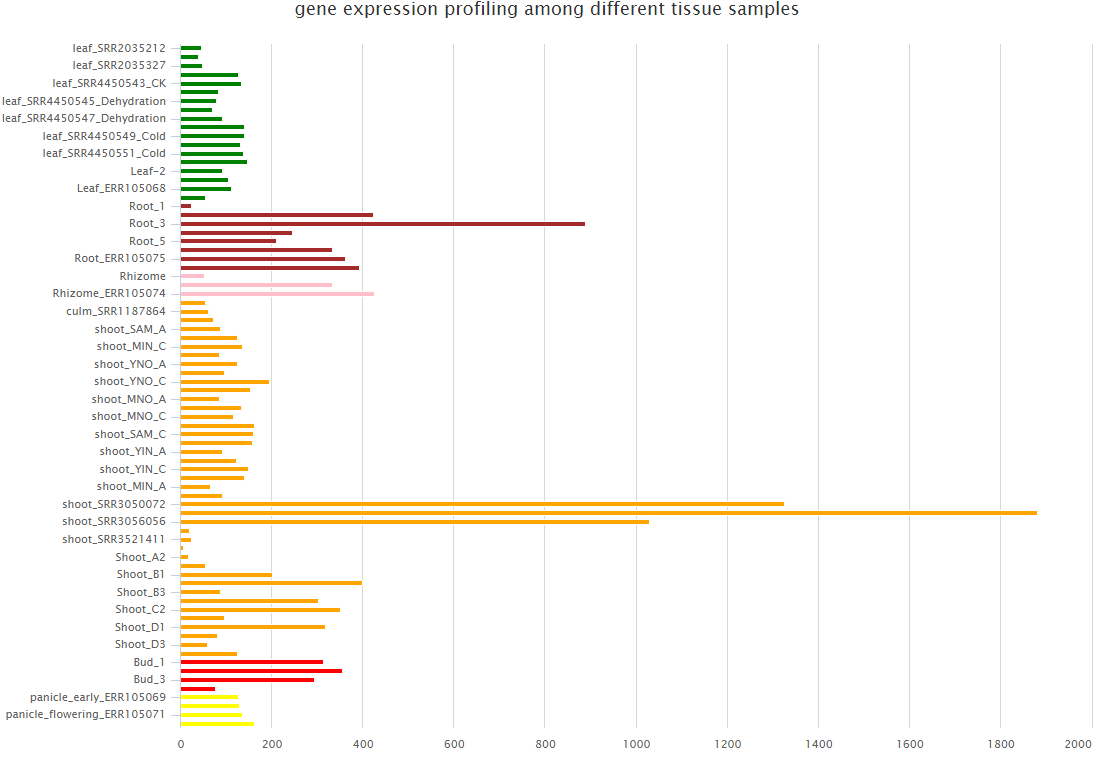 |
| AT4G05320(UBQ10; ubiquitin family protein) | PH02Gene03180.t1 | 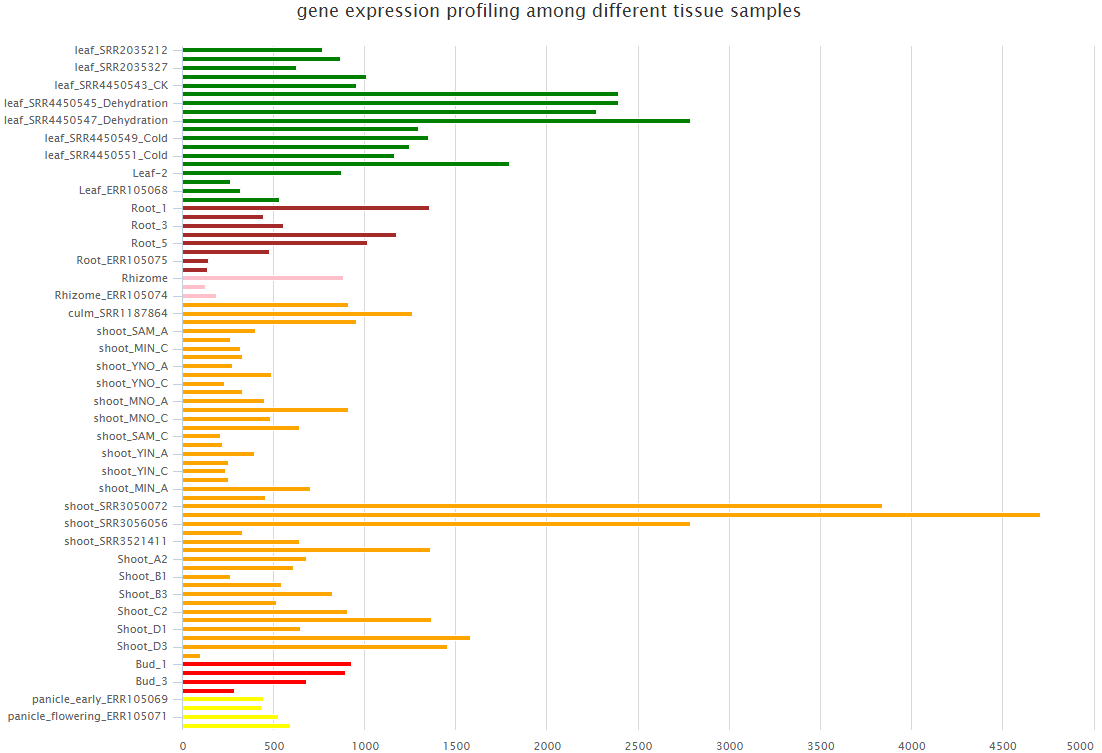 |
| AT5G60390(EF-1a4, GTP binding Elongation factor Tu family protein) | PH02Gene00084.t1 | 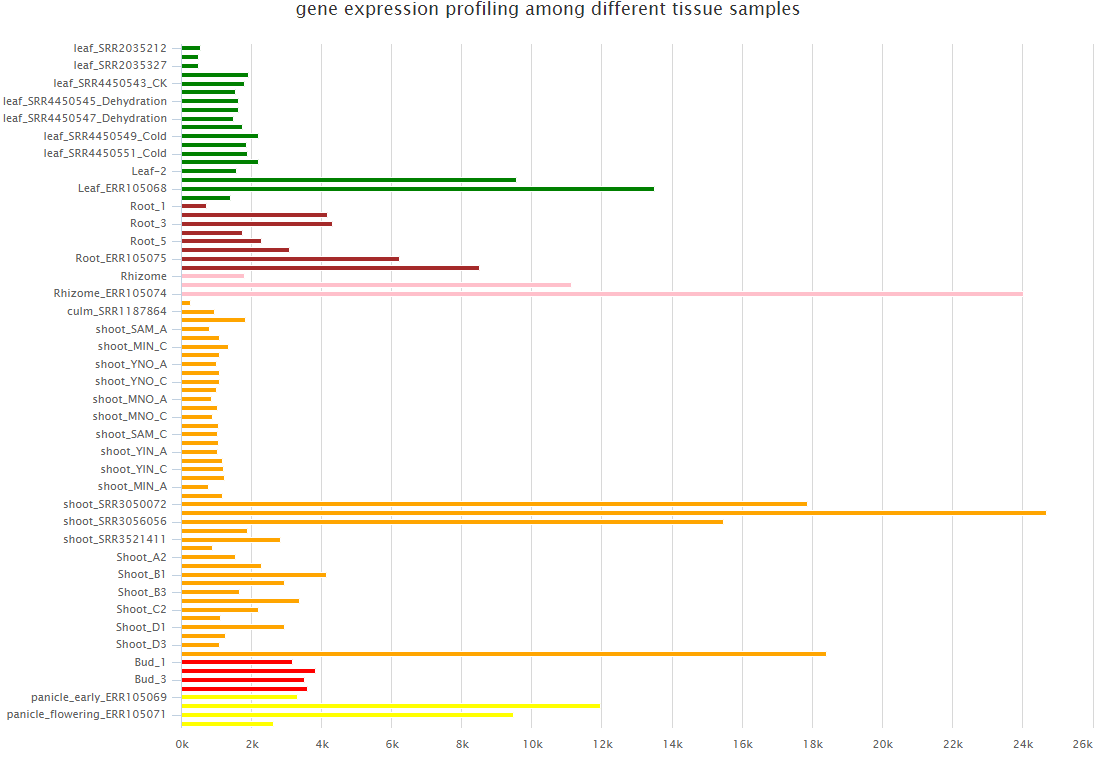 |
| At1g29330  (ERD2;  ER lumen protein retaining receptor family protein) | PH02Gene24865.t1 | 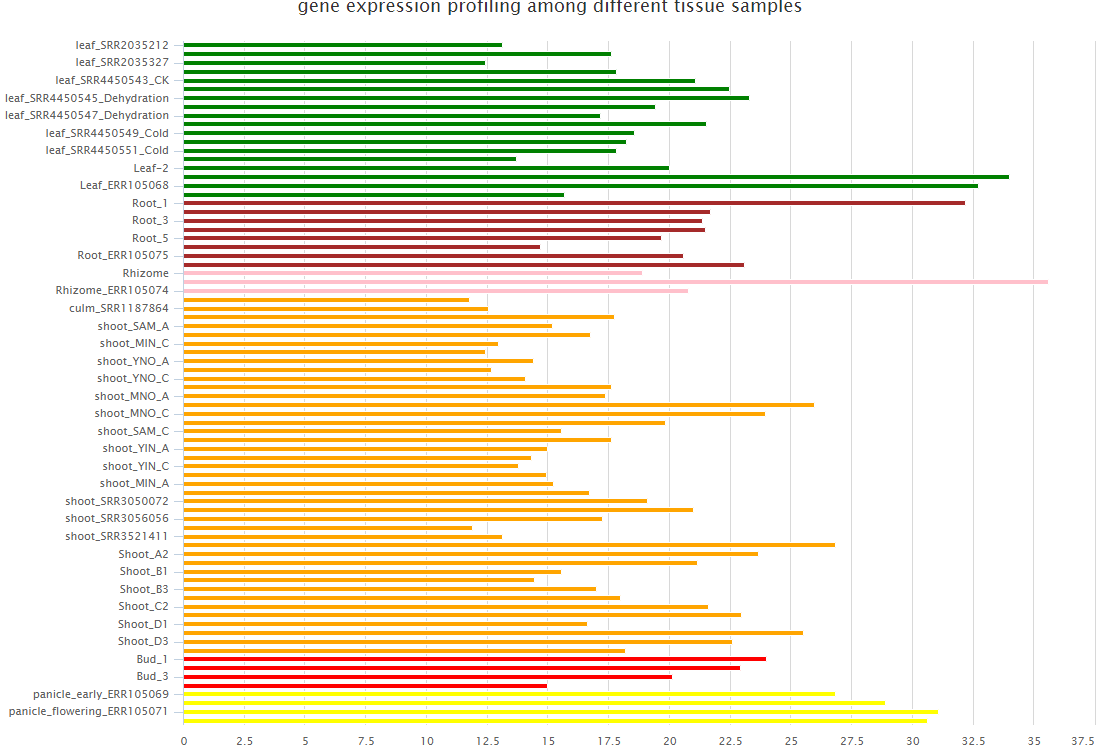 |
| p24δ3 (At1g09580; putative P24 ER-Golgi-trafficking GPI-anchor protein cargo receptor) | PH02Gene02587.t1 | 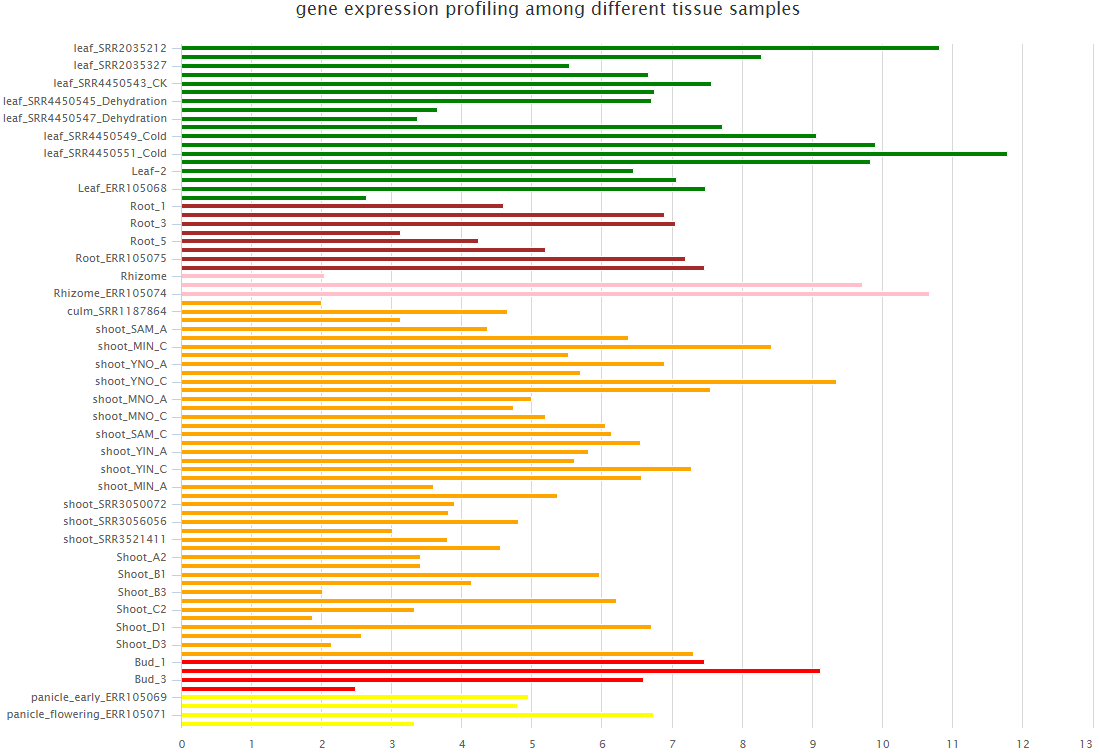 |
| p24δ4  (At1g57620; putative P24 ER-Golgi-trafficking GPI-anchor protein cargo receptor) |  | NA |
| p24δ5  (At1g21900; putative P24 ER-Golgi-trafficking GPI-anchor protein cargo receptor) |  | NA |
| p24δ6  (At3g10780; putative P24 ER-Golgi-trafficking GPI-anchor protein cargo receptor) |  | NA |
| p24δ7  (At1g14010; putative P24 ER-Golgi-trafficking GPI-anchor protein cargo receptor) | PH02Gene18174.t1 | 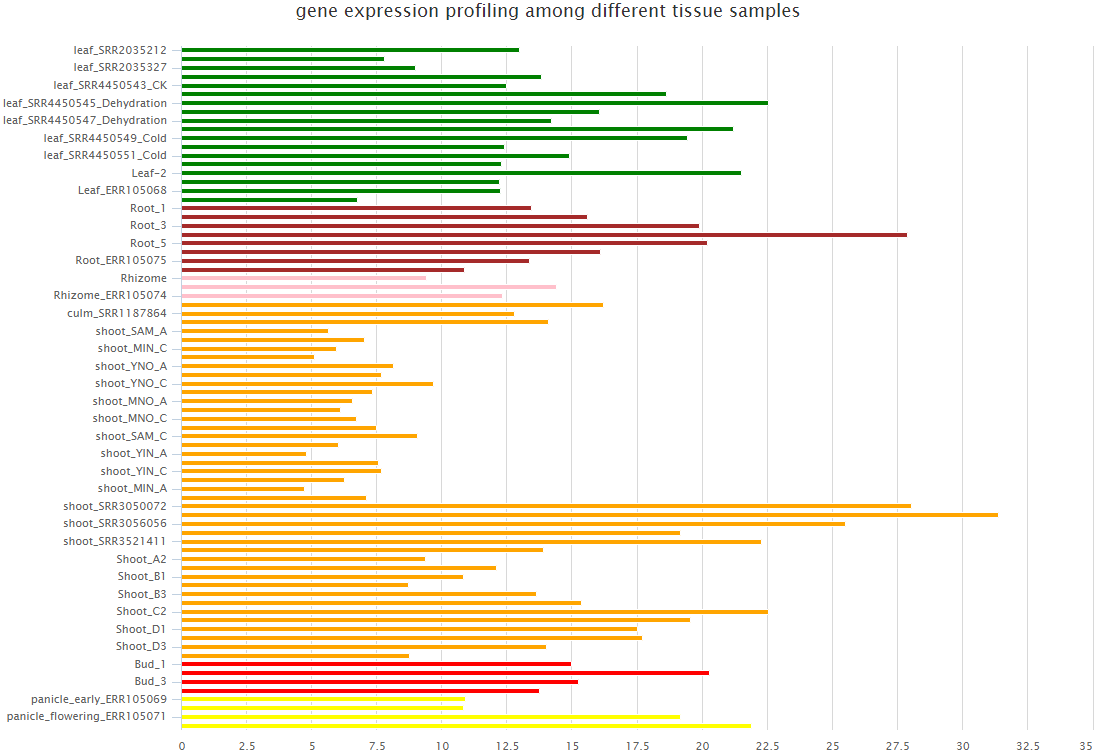 |
| p24δ8  (At2g03290; putative P24 ER-Golgi-trafficking GPI-anchor protein cargo receptor) |  | NA |
| p24δ9  (At1g26690; putative P24 ER-Golgi-trafficking GPI-anchor protein cargo receptor) | PH02Gene41513.t1 | 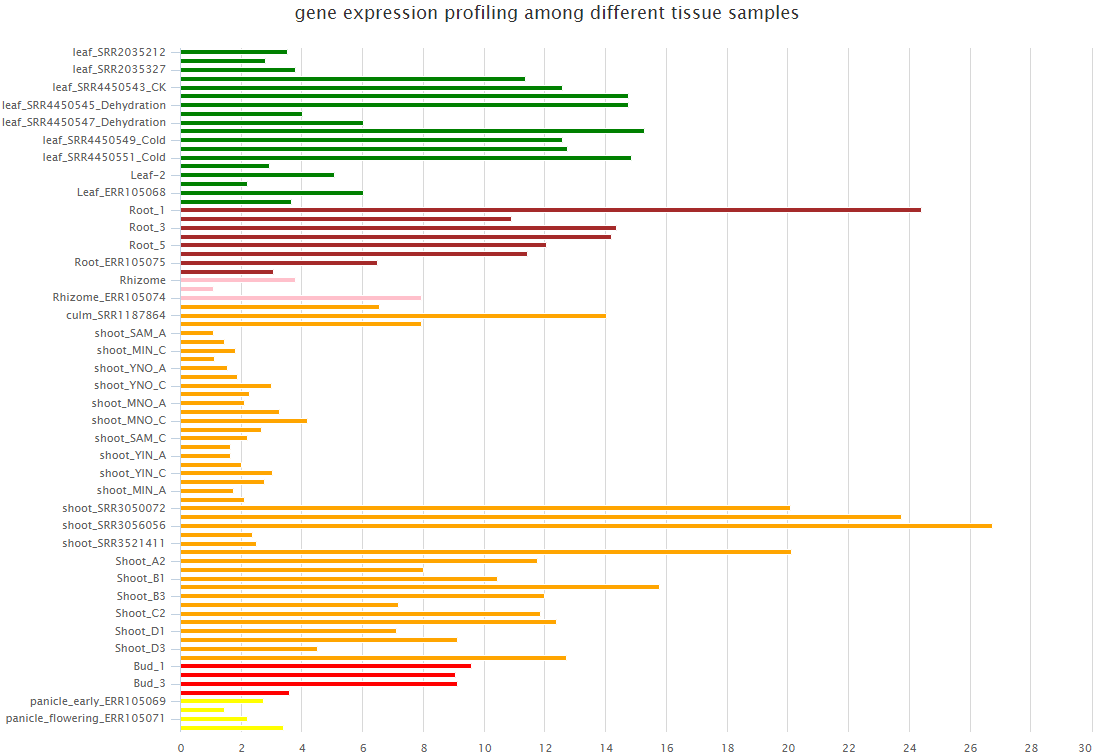 |
| p24δ10  (At1g69460; putative P24 ER-Golgi-trafficking GPI-anchor protein cargo receptor) |  | NA |
| p24δ11  (At3g29070; putative P24 ER-Golgi-trafficking GPI-anchor protein cargo receptor) |  | NA |
| AT1G10950  (EMP12;  transmembrane 9 superfamily member) | PH02Gene43095.t1 | 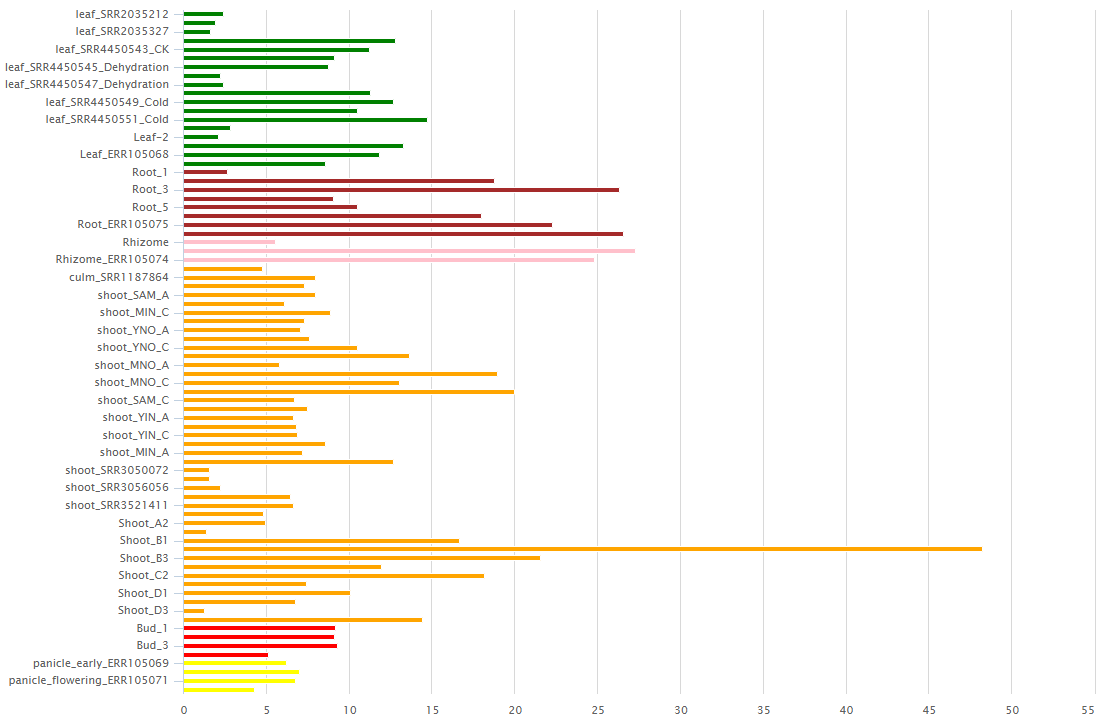 |
| AT4G31490  (β-COP; Putative EMP12 regulator) | PH02Gene23515.t1 | 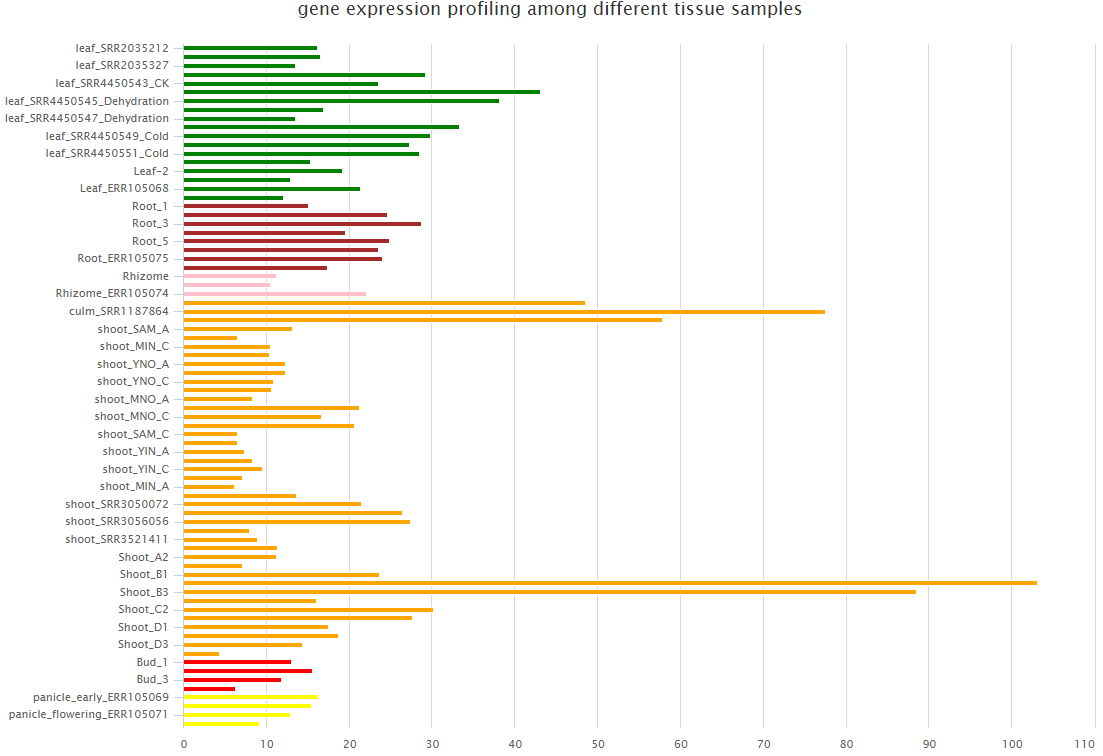 |
| AT1G30630  (ε-COP;  Putative EMP12 regulator) | PH02Gene01759.t1 | 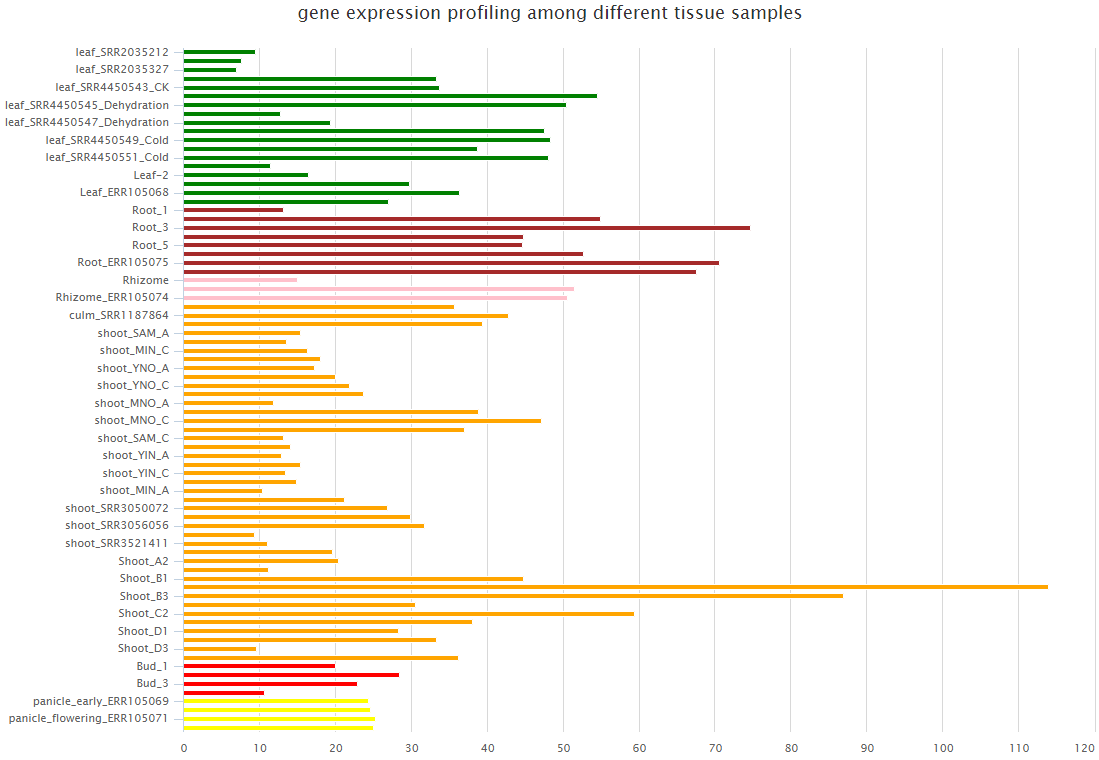 |
| At5g61790  (CNX1; calreticulin precursor protein) | PH02Gene29370.t1 | 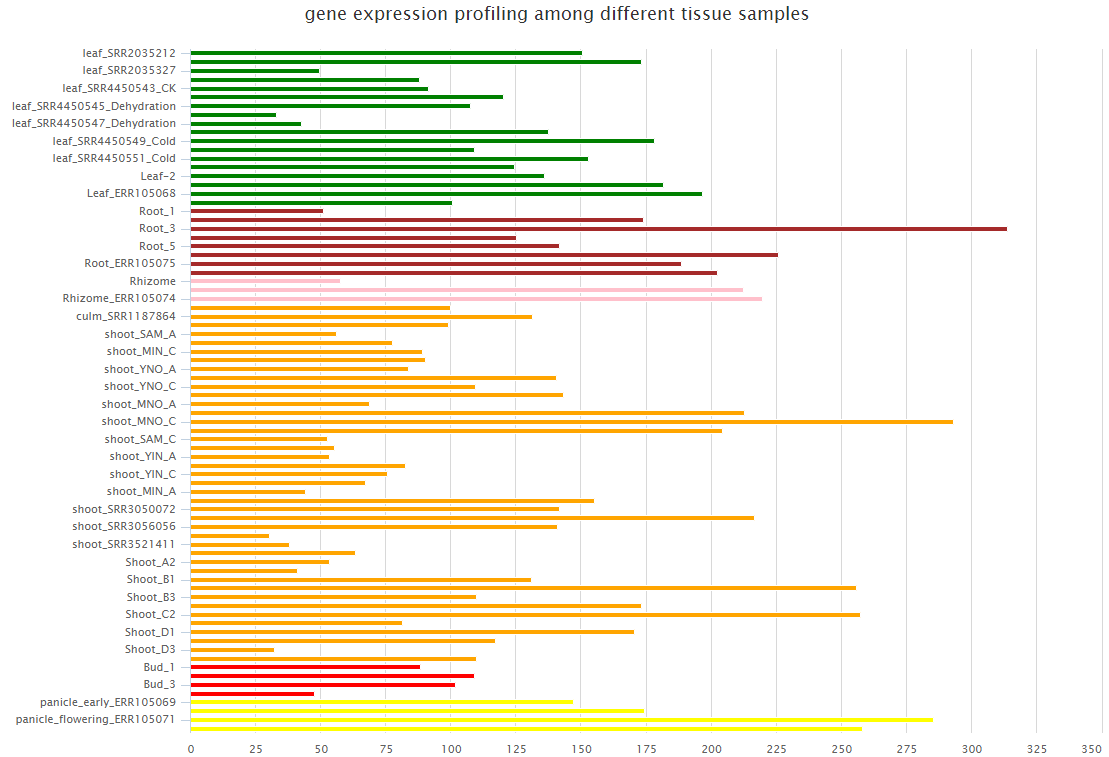 |
| AT5G60360 (AtAleurain) | PH02Gene03034.t1 | 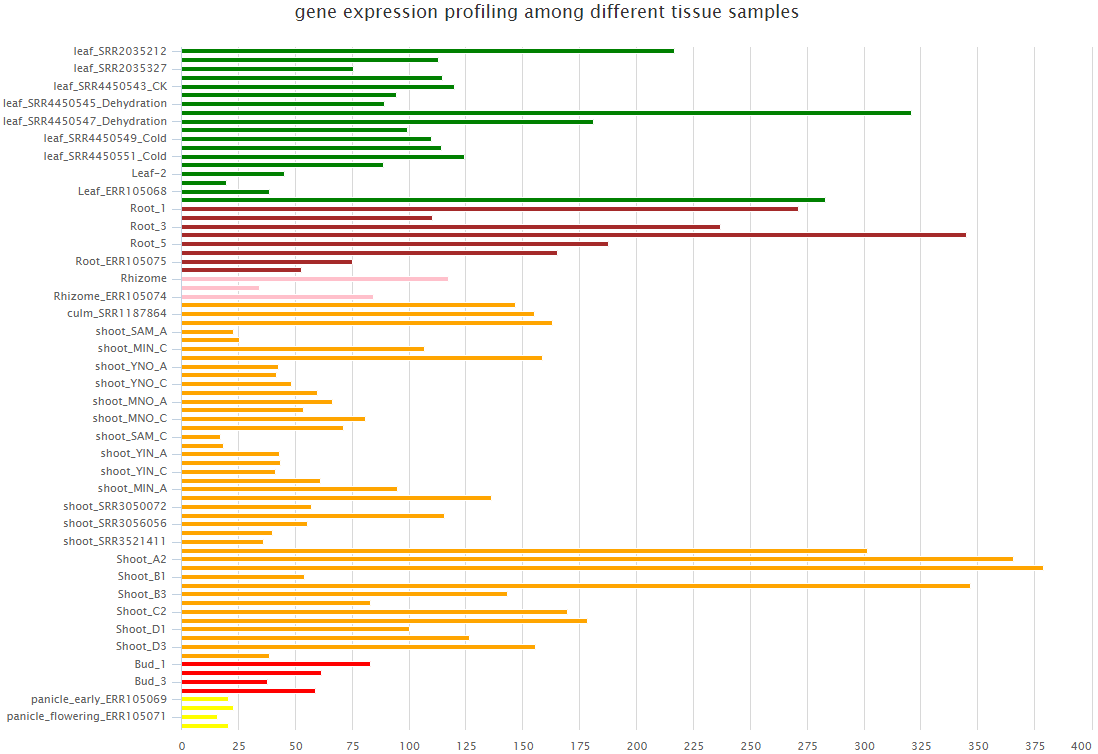 |
| AT3G52850  (AtVSR1)  vacuolar-sorting receptor precursor | PH02Gene12415.t1 | 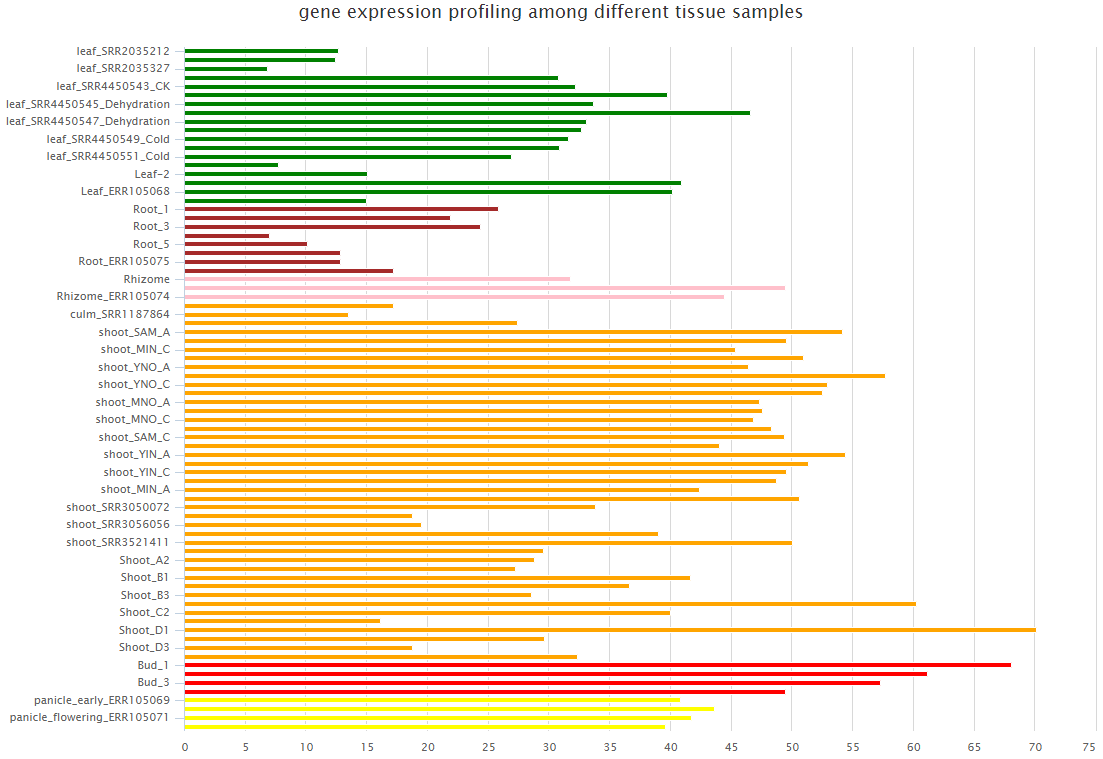 |

^*^The gene expression data was achieved from the BambooNET database (http://bioinformatics.cau.edu.cn/bamboo/index.html) (Ma et al., 2018). The expression level of homologues of internal control genes (actin7, UBQ10, and EF-1a4) and organelle marker genes (CNX, Aleurain, and VSR) were also shown for comparison. “NA” means the expression of moso bamboo homologue is not detected.
