# Supplementary material for: Organelle Visualization With Multicolored Fluorescent Markers in Bamboo
Source: Front Plant Sci. 2021 Apr 15;12:658836. doi: 10.3389/fpls.2021.658836 (PMC8081836; doi:10.3389/fpls.2021.658836)
Supplement: Supplementary Table 3 — Detailed information of primer sequences for each construct. [file Table_3.DOCX]

**Supplementary Table 3. Detailed information of primer sequences for each construct.**

| Construct name | Primer name and sequence (from 5’ start to 3’ end) |
| --- | --- |
| PeCNX-(X)FP | JA-206-PeCNX-XFP-XbaI-F  GGG TCT AGA ATG GAT GTC ATT GAT AAG GGA GAG AAG C  JA-207-PhCNX-XFP-XhoI-R  GGG CTC GAG TGT CTC CCT TCG GGA TCT CCG GC |
| PeManI-(X)FP | JA-208-PeManI-XFP-XbaI-F  GGG TCT AGA ATG GCT CGT CGG TCG TCA TCC  JA-209-PhManI-XFP-BamHI-R  GGG GGA TCC AAT CTC CGA CTC GTA CTC GCG G |
| PeERD2-(X)FP | JA-210-PeERD2-(X)FP-XbaI-F  GGG TCT AGA ATG AAC GCC TTT CGC TTC CT  JA-211-PeERD2-(X)FP-BamHI-R  GGG GGA TCC AGC AGG CAA CTC AAG CTT CAC |
| (X)FP-PeMEMB12 | JA506-XFP-PeMEMB12-XbaI-F  GGG TCT AGA GCT CGC AGC TCC TCT CGT ATG  JA-340-XFP-PeMEMB12-XhoI-R  GGG CTC GAG CTA ATG CGT CCA CCG CCA AAA |
| RFP-PeSYP61 | JA-212-XFP-PeSYP61-SpeI-F  GGG ACT AGT ATG AGC TCC GCG CAA GAT CCA TTC  JA-213-XFP-PeSYP61-XhoI-R  GGG CTC GAG TTA TGT CAA GAA CAC CAA AAC GAA AAG TAT AAT GAA CAG A |
| (X)FP-PeSYP42 | \| JA-347-XFP-PeSYP42-XbaI-F  GGG TCT AGA ATG TTG CAA AAG GCT GAG AGG AC  JA-348-XFP-PeSYP42-SacI-R  GGG GAG CTC TCA GAA AAG TAT CTC CTT CAA AAT CAG G \| \| --- \| |
| (X)FP-PeVTI12-FL | \| JA-351-XFP-PeVTI12-XbaI-F  GGG TCT AGA ATG AGC GAG GTA TTC GAG GGC  JA-352-XFP-PeVTI12-XhoI-R  GGG CTC GAG TTA ATG AGT GAG CTT AAA GTA CAG TAT GAG AAG \| \| --- \| |
| (X)FP-PeVTI12 | JA-648-XFP-PeVTI12-XbaI-F  GGG TCT AGA ATG GGT GTG TCA ATT CTT CAG GA  JA-352-XFP-PeVTI12-XhoI-R  GGG CTC GAG TTA ATG AGT GAG CTT AAA GTA CAG TAT GAG AAG |
| (X)FP-PeARA7 | JA-216-XFP-PeRabF2b-XbaI-F  GGG TCT AGA ATG GCA GCC AAC GCC GG  JA-217-XFP-PeRabF2b-SacI-R  GGG GAG CTC TCA AGA GCA GCA TGA AGA AGC GC |
| (X)FP-PeVSR1 | JA-220-SP-XFP-PeVSR-XbaI-F  GGG TCT AGA ATG GAC ACT TGT ATC AAC AAA GAG GCT ACC TC  JA-221-SP-XFP-PeVSR-SacI-R  GGG GAG CTC TCA AAT ATC ATC AGC GTG CGC AAC ATG A |
| (X)FP-PeVAMP711 | JA507-XFP-PeVAMP711-XbaI-F  GGG TCT AGA GAT CGT TTG GAA CTG CTG GTT GAC A  JA-227-XFP-PeVAMP711-SacI-R  GGG GAG CTC TTA CCG GAT ACA AGT TGG TAA GGT GAA GC |
| (X)FP-PeVIT1 | JA-230-XFP-PeVIT1-XbaI-F  GGG TCT AGA ATG GTG TTG GAG AGC GGC AAG AAG  JA-231-XFP-PeVIT1-SacI-R  GGG GAG CTC CTA GAT GGA CTG CAC GGC CTT GGC |
| Pealeurain-(X)FP | JA-234-Pealeurain-XFP-XbaI-F  GGG TCT AGA ATG GCC CGC CGC TGC  JA-235-Pealeurain-XFP-BamHI-R  GGG GGA TCC GGA CTC GAG CGC GGA GGC |
| (X)FP- PeVAMP721 | JA505-XFP-PeVAMP722-XbaI-F  GGG TCT AGA CAA GAT TTC AGA CAG CAG GGT ACA  JA-239-(X)FP- PeVAMP722-SacI-R  GGG GAG CTC TCA CTT GCA TTT GAA GCC ATG GCA CAC |
| (X)FP-PePIP1;1 | JA486-XFP-PePIP1-XbaI-F  GGG TCT AGA ATG GAG GGG AGG GAG GAG GAC  JA487-XFP-PePIP1-XhoI-R  GGG CTC GAG TTA AGA TCT GCT CTT AAA TGG TAT TGC |
| PeMADS5-GFP | JA503-PeMADS5-XFP-XbaI-F  GGG TCT AGA ATG GCG CGG GAG CGG CGG GA  JA504-PeMADS5-XFP-XhoI-R  GGG CTC GAG CTT CCA TGC AAC ACA AGG CAA CC |
| (X)FP-PePIP2;3 | JA513-XFP-PePIP2-3-XbaI-F  GGG TCT AGA ATG GCG AAA GAC ATT GAG GCG TC  JA514-XFP-PePIP2-3-XhoI-R  GGG CTC GAG TTA GGC GTT GCT CCG GTA GGA CC |
| Cerulean-PeVSR1-FL | JA-511-PeVSR-XbaI-F  GGGTCTAGAATGAGGGGACCGGTGCTGTGGGC JA-221-SP-XFP-PhVSR-SacI-R  GGGGAGCTCTCAAATATCATCAGCGTGCGCAACATGA |
